# Genomic Imprinting in the *Arabidopsis* Embryo Is Partly Regulated by PRC2

**DOI:** 10.1371/journal.pgen.1003862

**Published:** 2013-12-05

**Authors:** Michael T. Raissig, Marian Bemer, Célia Baroux, Ueli Grossniklaus

**Affiliations:** Institute of Plant Biology & Zürich-Basel Plant Science Center, University of Zürich, Zürich, Switzerland; University of Minnesota, United States of America

## Abstract

Genomic imprinting results in monoallelic gene expression in a parent-of-origin-dependent manner and is regulated by the differential epigenetic marking of the parental alleles. In plants, genomic imprinting has been primarily described for genes expressed in the endosperm, a tissue nourishing the developing embryo that does not contribute to the next generation. In *Arabidopsis*, the genes *MEDEA* (*MEA*) and *PHERES1* (*PHE1*), which are imprinted in the endosperm, are also expressed in the embryo; whether their embryonic expression is regulated by imprinting or not, however, remains controversial. In contrast, the *maternally expressed in embryo 1* (*mee1*) gene of maize is clearly imprinted in the embryo. We identified several imprinted candidate genes in an allele-specific transcriptome of hybrid *Arabidopsis* embryos and confirmed parent-of-origin-dependent, monoallelic expression for eleven maternally expressed genes (MEGs) and one paternally expressed gene (PEG) in the embryo, using allele-specific expression analyses and reporter gene assays. Genetic studies indicate that the *Polycomb* Repressive Complex 2 (PRC2) but not the DNA *METHYLTRANSFERASE1* (*MET1*) is involved in regulating imprinted expression in the embryo. In the seedling, all embryonic MEGs and the PEG are expressed from both parents, suggesting that the imprint is erased during late embryogenesis or early vegetative development. Our finding that several genes are regulated by genomic imprinting in the *Arabidopsis* embryo clearly demonstrates that this epigenetic phenomenon is not a unique feature of the endosperm in both monocots and dicots.

## Author Summary

In most cells nuclear genes are present in two copies, with one maternal and one paternal allele. Usually, the two alleles share the same fate regarding their activity, with both copies being active or both being silent. An exception to this rule are genes that are regulated by genomic imprinting, where only one allele is expressed and the other one remains silent depending on the parent it was inherited from. The two alleles are equal in terms of their DNA sequence but carry different epigenetic marks distinguishing them. Genomic imprinting evolved independently in mammals and flowering plants. In mammals, genes regulated by genomic imprinting are expressed in a wide range of tissues including the embryo and the placenta. In plants, genomic imprinting has been primarily described for genes expressed in the endosperm, a nutritive tissue in the seed with a function similar to that of the mammalian placenta. Here, we describe that some genes are also regulated by genomic imprinting in the embryo of the model plant *Arabidopsis thaliana*. An epigenetic silencing complex, the *Polycomb* Repressive Complex 2 (PRC2), partly regulates genomic imprinting in the embryo. Interestingly, embryonic imprints seem to be erased during late embryo or early seedling development.

## Introduction

Genes regulated by genomic imprinting are expressed preferentially from one allele in a parent-of-origin-dependent manner. The two alleles do not differ in their DNA sequence, but rather they are differentially marked by epigenetic modifications. In mammals, imprint establishment occurs in a sex-specific manner during gamete development [1]. The parent-of-origin-specific “imprint” is retained or interpreted after fertilization, such that the alleles can be distinguished and differentially expressed during subsequent development. The imprints are erased in the primordial germ cell lineage, which will develop into the gametes, and are reestablished according to the sex of the germ line during gametogenesis [2], [3].

Genomic imprinting evolved both in placental mammals and in flowering plants. While genes can be imprinted in both the embryo and the placenta in mammals, and even in adult tissues [2], [4], genomic imprinting in plants was primarily described for genes expressed in the endosperm, the triploid nourishing tissue that develops upon fertilization of the diploid central cell [5], [6]. The triploid endosperm does not contribute to the next generation and, therefore, there is no requirement to erase and reset parental imprints. To date, only four plant genes have been proposed to show parent-of-origin-dependent, monoallelic expression in both the embryo and the endosperm, but only for the maize gene *maternally expressed in embryo1* (*mee1*) was this unambiguously demonstrated [7]. Although only maternal transcripts of the *Os10g05750* gene were detected in rice embryos, it is not clear whether these stem from expression in the egg cell or are monoallelically expressed *de novo* after fertilization, as is necessary for classification as an imprinted gene [8]. In *Arabidopsis thaliana*, the *Polycomb* group gene *MEDEA* (*MEA*) and its target, the MADS-box gene *PHERES1* (*PHE1*), are both imprinted in the endosperm and show embryonic expression [9]–[11]; but it remains controversial whether embryonic expression is imprinted or not (reviewed in [10]).

The regulation of genomic imprinting in mammals is complex and involves DNA methylation, histone modifications, and non-coding RNAs [2], [12], [13]. In *Arabidopsis*, DNA methylation and *Polycomb* Repressive Complex 2 (PRC2)-mediated trimethylation of histone 3 lysine 27 (H3K27me3) are involved in the regulation of some imprinted loci in the endosperm. The maintenance DNA-methyltransferase METHYLTRANSFERASE1 (MET1) and the DNA-glycosylase DEMETER (DME) act antagonistically to regulate imprinting at the *MEA*, *FLOWERING WAGENINGEN* (*FWA*), *FERTILIZATION INDEPENDENT SEED2* (*FIS2*), and *MATERNALLY EXPRESSED PAB C-TERMINAL* (*MPC*) loci [14]–[19]. *DME* is preferentially expressed in the central cell and removes DNA-methylation marks on maternal alleles [14], [17], which, however, might not directly define the imprinting status at all loci [20]. In maize, most imprinted genes analyzed in detail are differentially methylated in the endosperm [7], [21]–[23], which is already established in the gametes for some loci but not for others, indicating the existence of additional, primary imprinting marks other than DNA-methylation [7], [21]. The regulation of imprinted *MEA* expression by DME and MET1 is also indirect and it is not the presence or absence of DNA methylation that leads to differential expression of the two parental *MEA* alleles [20]. Finally, the regulation of imprinted expression of *MEA, PHE1, FORMIN-HOMOLOGUE 5* (*AtFH5*), and some other loci involves additionally or exclusively the repressive action of PRC2 [9], [17], [24]–[27]. Recent studies identified many novel candidate imprinted genes in *Arabidopsis*, maize, and rice using systematic, genome-wide transcriptome screens on seed tissues [8], [27]–[32]. In *Arabidopsis* alone, the total number of imprinted genes increased from 12 to more than 300 potentially imprinted genes [27]–[30].

In this study, we show that genomic imprinting is not restricted to the endosperm in *Arabidopsis*, and describe parent-of-origin-dependent, monoallelic expression in the *Arabidopsis* embryo. We identified 80 potentially imprinted genes from a parent-of-origin-specific embryonic transcriptome [33] and confirmed eleven MEGs and one early PEG using allele-specific expression analyses of parental transcripts and reporter gene assays. Furthermore, we found that PRC2 is involved in maintaining the imprinted expression pattern at some of these loci. In contrast to imprinting in the endosperm, imprinted expression in the embryo requires the erasure and resetting of the imprinting marks between generations. Interestingly, the MEGs and the PEG are expressed from both alleles in seedlings, suggesting that the imprints are erased during late embryogenesis or early seedling development.

## Results

### In-Depth Analysis of the Hybrid Embryonic Transcriptome Reveals Monoparentally Expressed Genes

To study the global parental contributions to the embryonic transcriptome and its regulation, embryonic samples were previously generated from hybrid embryos at the 2–4 cell and globular stage [33]. The hybrid embryos were derived either from a cross between Landsberg *erecta* (Ler) and Columbia-0 (Col-0), or from a cross between the *kryptonite* (*kyp*) mutant (Ler) and Col-0. Subsequent high-throughput sequencing of the generated cDNA libraries allowed the identification of allele-specific transcripts based on single nucleotide polymorphisms (SNPs) between the accessions [33]. For this study, we identified potentially imprinted transcripts in the embryo, for which only one parental allele was sequenced in all samples, in the 2–4 cell samples only, or in the globular samples only ([33] for details see Material and Methods). We included only genes that were not deregulated in the *kyp/KYP* x Col-0 sample, assuming that *KYP* is not a major regulator of genomic imprinting, but rather regulates the parental contributions at the genome-wide level [33]. This procedure yielded 50 potential maternally expressed genes (MEGs) and 30 potential paternally expressed genes (PEGs) in the *Arabidopsis* embryo (Table S1). A recent study analyzing a hybrid embryonic transcriptome in *Arabidopsis* [34] also detected the presence of monoallelic gene expression in the *Arabidopsis* embryo. The authors describe 77 maternally and 45 paternally contributed transcripts in at least one embryonic stage tested. Finally, we chose 18 MEG candidates and six PEG candidates that are highly expressed in the embryo [33] but are absent from the gametes [35], [36], suggesting *de novo* expression, and analyzed them in detail (i.e. allele-specific expression and reporter gene analysis; Table S1).

### Monoallelic Gene Expression in the 2–4 Cell and the Globular Embryo

To confirm the identified MEGs and PEGs, we produced new embryonic cDNA libraries by crossing the Col-0 and the Ler accessions reciprocally. Embryos were isolated at the 2–4 cell embryo stage (∼2.5 days after pollination (DAP)) and at the globular embryo stage (∼4 DAP). We sampled two biological replicates of each cross and stage (8 samples in total), extracted total RNA and amplified a cDNA library (see Material and Methods). The cDNA samples from hybrid embryos were subsequently used to amplify the polymorphic, SNP-containing sequence of potentially imprinted transcripts by RT-PCR, and products were assessed for their parent-of-origin by Sanger sequencing. As a control, we performed allele-specific expression analysis of a polymorphic gene that is expressed from both parental alleles (*AT1G02780*, *EMBRYO DEFECTIVE 2386*, [33]). We readily detected both parental nucleotides at the polymorphic site in all the samples analyzed (Figure S1A), confirming that this method is suitable to detect biallelic gene expression. Importantly, we verified that all assays used in this study amplify both parental alleles with equal efficiency. To this aim we performed PCR and Sanger sequencing on genomic DNA from reciprocal F1 hybrid seedlings (Col-0 x Ler and Ler x Col-0, respectively; Figure S1B and Figure S2). We assessed also the quantitative nature of the Sanger sequencing approach by performing the different assays with various mixtures of Col-0 and Ler genomic DNA. All assays showed a good correlation between the allelic ratios and the SNP-signal, illustrating that the Sanger approach is valid to estimate allele contributions (Figure S3). In conclusion, all assays used in this study amplified both alleles with equal efficiency and, thus, do not introduce a technical bias towards one allele.

**Figure 1:**
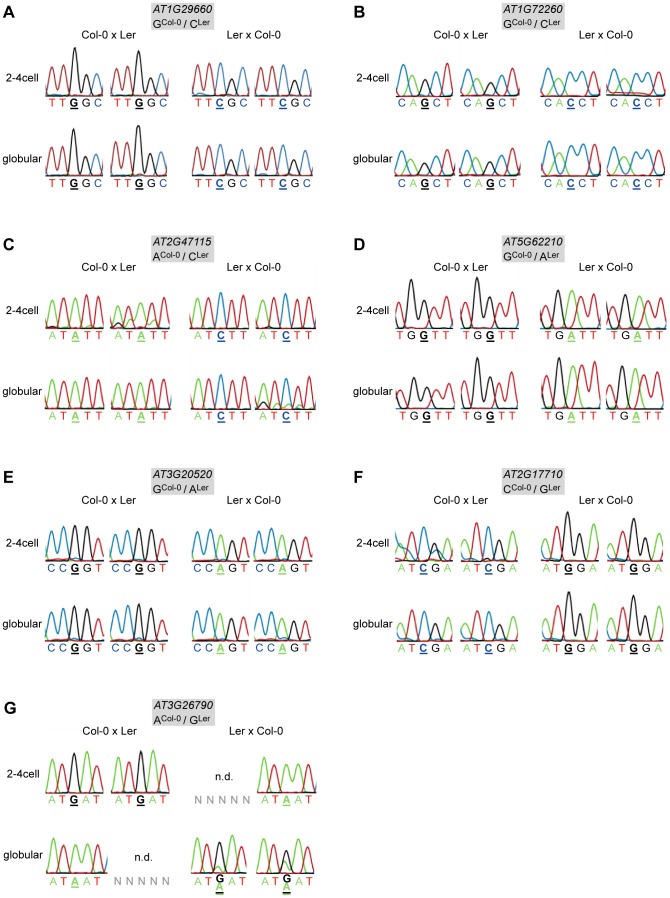
Allele-specific expression analysis of MEGs and PEGs. Reciprocal hybrid embryos were isolated at 2.5 DAP (2–4 cell embryos) and at 4 DAP (globular embryos) and allele-specific expression was analyzed by RT-PCR and Sanger sequencing. The direction of the cross is indicated on top of each panel, the embryonic stage on the left. Two replicates were analyzed for each stage and cross, which is represented by two individual sequencing chromatograms. The analyzed gene and the polymorphism between Col-0 and Ler are indicated in the grey box atop each panel. Furthermore, the polymorphic nucleotide is displayed in bold and underlined below each chromatogram. n.d. indicates that the transcript could not be amplified from the specific embryo replicate sample. (A) *AT1G29660*. (B) *AT1G72260*. (C) *AT2G47115*. (D) *AT5G62210*. (E) *AT3G20520*. (F) *AT2G17710*. (G) *FUS3* (*AT3G26790*).

We then performed allele-specific expression analyses on the selected 18 candidate MEGs and six candidate PEGs (Table S1). For eleven of the 18 candidate MEGs we could sequence only the maternal allele in all crosses, stages, and replicates analyzed (*AT1G29660, AT1G72260, AT2G47115, AT5G62210, AT3G20520, AT2G17710, AT3G21500, AT2G01520, AT1G20680, AT5G51950, AT1G29050*; Figure 1A–1F and Figure S4A–S4E). Because 9 of the 11 genes showed no detectable levels in the egg cell transcriptome, our results strongly suggest that they may be regulated by genomic imprinting in the *Arabidopsis* embryo ([35], [36], Table S1). Two of the 18 candidate MEGs showed biallelic expression in one 2–4 cell replicate sample and were therefore excluded from further analyses (*AT3G44260* and *AT5G52060*, Figure S4F and S4G). However, the maternal signal was much higher in these replicates, and both genes showed complete monoallelic expression at the globular stage, suggesting that they may also be regulated by genomic imprinting. From the remaining five MEGs, four genes could not be amplified at all from the embryonic cDNA libraries, and one gene was lost when a highly stringent washing procedure was applied to the embryos before RNA extraction and amplification (see below, Table S1), suggesting that they are not or only weakly expressed in the embryo.

Of the six analyzed candidate PEGs, only *FUSCA3* (*FUS3, AT3G26790*) showed consistent monoallelic expression at the 2–4 cell stage, but not at the globular stage, at which expression was either maternal or biallelic, depending on the direction of the cross (Figure 1G). In two replicates, *FUS3* could not be detected at all, which might indicate that *FUS3* is expressed at a low level in the embryo; even below detection level in some samples. Two other candidate PEGs were found to be expressed from both parents (*MEIDOS* (*AT2G20160*) and *AT1G63260*; Figure S5), while the three remaining candidate PEGs could not be detected at all in our embryonic cDNA libraries (Table S1).

In summary, we could confirm eleven MEGs and one PEG that show parent-of-origin-dependent, monoallelic gene expression in the embryo. For nine MEGs and the PEG no transcripts were found in the gametes suggesting *de novo* expression and, therefore, true imprinted expression in the embryo [35], [36]. One MEG, *AT1G72260*, is expressed in the gametes already, and another MEG, *AT2G47115*, is not represented on the microarrays, such that no statement can be made about *de novo* transcription in the embryo [35], [36]. Nevertheless, these findings show that genomic imprinting in the embryo is more widespread than commonly thought and that imprinted expression of the maize *mee1* gene is not an exceptional case.

### Monoallelic Expression Patterns Are Not Due to Sporophytic or Endosperm Contamination

The embryo is surrounded by the triploid endosperm, and both embryo and endosperm are embedded in the maternal, sporophytic seed coat. Therefore, isolating embryos devoid of debris from surrounding sporophytic tissues and careful control of maternal tissue contamination is an important issue [34]. While the PEG cannot be derived from seed coat contamination, substantial contamination with maternal sporophytic tissue could explain the observed maternal expression patterns of the confirmed MEGs. Our initial samples were prepared from embryos diluted in a large volume and additionally washed one time. Although all samples were devoid of visible debris at collection, we produced two additional embryonic cDNA libraries to rule out the possibility of sporophytic contamination: following reciprocal crosses between Col-0 and Ler, we isolated 2–4 cell embryos, but washed them six times (6×) instead of one time (1×), a procedure that should result in the removal of all possible non-embryonic transcripts but may also degrade embryonic transcripts.

**Figure 2:**
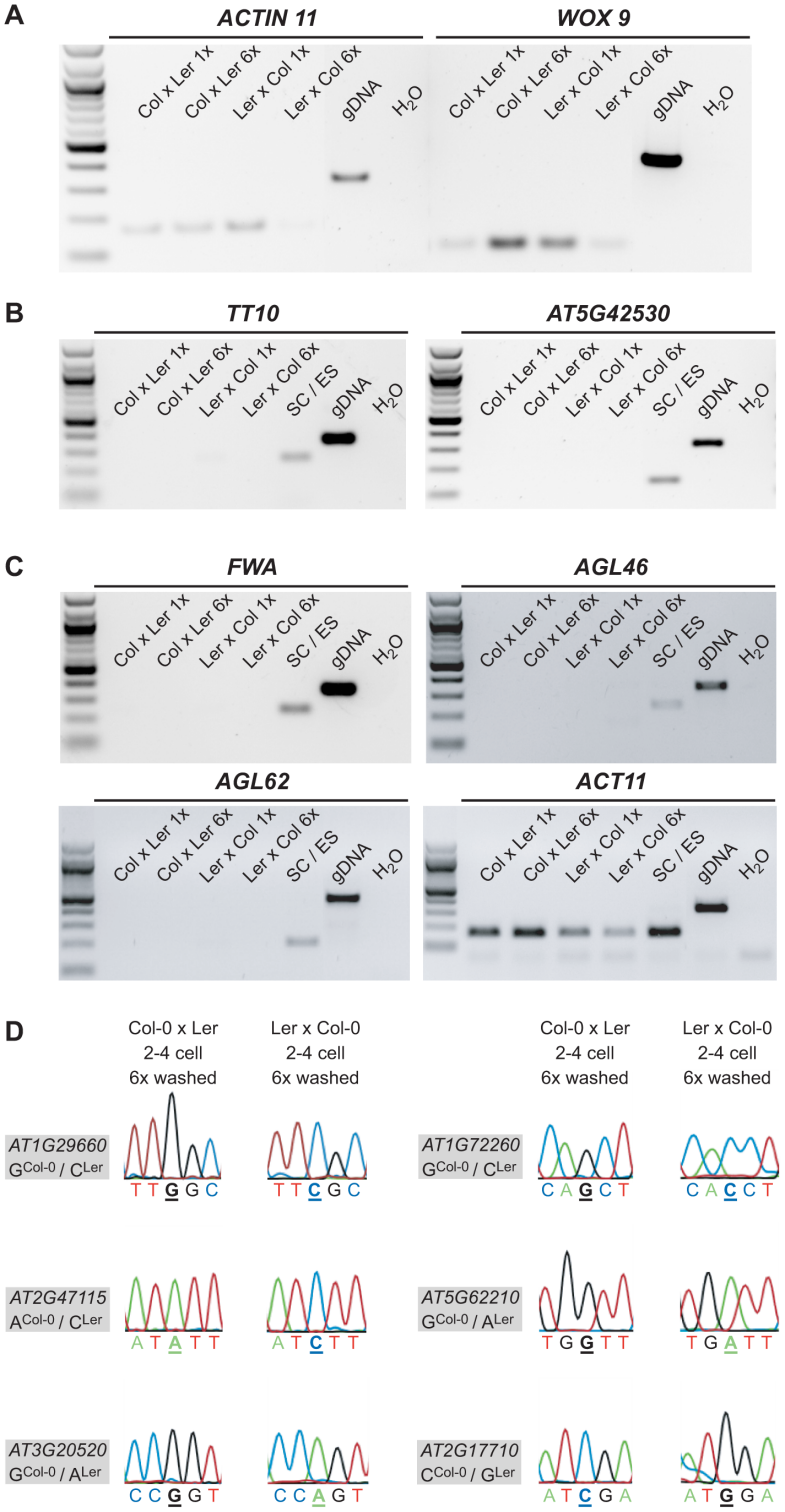
Quality, purity and allele-specific expression analyses of extensively washed embryonic control samples. (A) RT-PCR amplifying *ACT11* and *WOX9*, an embryo-specific gene, from 1× washed and 6× washed reciprocal, embryonic cDNA libraries (2–4 cell stage) using 32 PCR cycles. Genomic DNA was used as positive and water as negative control. (B) RT-PCR amplifying *TT10* and *AT5G42530*, two seed coat-specific genes ([37] and our own analysis based on [33]), from 1× washed and 6× washed reciprocal, embryonic cDNA libraries (2–4 cell stage) and a combined seed coat/endosperm sample using 28 and 32 PCR cycles, respectively. Genomic DNA was used as positive and water as negative control. (C) RT-PCR amplifying *FWA, AGL46* and *AGL62*, three endosperm-specific genes [16], [38], [39], from 1× washed and 6× washed reciprocal, embryonic cDNA libraries (2–4 cell stage) and a combined seed coat/endosperm sample using 28, 32 and 32 PCR cycles, respectively. Genomic DNA was used as positive and water as negative control. In addition, *ACT11* was amplified again including the combined seed coat and endosperm sample. (D) Allele-specific expression analysis of *AT1G29660*, *AT1G72260*, *AT2G47115*, *AT5G62210*, *AT3G20520*, and *AT2G17710* in the 6× washed embryonic cDNA libraries. The analyzed gene and the polymorphism between Col-0 and Ler are indicated in the grey box and the direction of the cross and the stage on top of the panel. The polymorphic nucleotide is displayed in bold and underlined below each chromatogram.

First, we assessed the quality of the 6× washed embryonic cDNA libraries compared to the 1× washed libraries by performing RT-PCR using primers amplifying *ACTIN 11* (*ACT11*) and *WUSCHEL-RELATED HOMEOBOX 9* (*WOX9*), an embryo-specific gene (Figure 2A). We amplified both control genes in the 1× and the 6× washed embryonic cDNA libraries derived from the Col-0 x Ler cross (Figure 2A). We could only weakly amplify *ACT11* and *WOX9* in the 6× washed Ler x Col-0 library (Figure 2A). This indicates a lower cDNA library quality of this sample, likely due to RNA degradation during the washes. Second, we confirmed the absence of seed coat and endosperm expressed genes, for which no embryo expression has been detected (based on literature, our own and available online embryo expression data) in our embryo samples by RT-PCR (Figure 2B and 2C). As seed coat markers, we tested *TRANSPARENT TESTA 10* (*TT10*; [37]) and *AT5G42530* (our own analysis based on [33]), and as endosperm markers, we tested *FWA* [16], *AGAMOUS-LIKE 46* (*AGL46*; [38]), and *AGAMOUS-LIKE 62* (*AGL62*; [39]). All genes showed distinct expression in a combined seed coat/endosperm sample, but were absent from all 1× and 6× washed embryo samples, suggesting that our previous washing regime is sufficient to remove non-embryonic transcripts.

We could readily detect the transcript of seven MEGs (*AT1G29660, AT1G72260, AT2G47115, AT5G62210, AT3G20520, AT2G17710, AT3G21500*) in both 6× washed 2–4 cell embryo samples and confirmed their monoallelic expression pattern by Sanger sequencing analysis (Figure 2D, Figure S6A). For the other four MEGs (*AT2G01520, AT1G20680, AT5G51950, AT1G29050*) we could only detect expression in the 6× washed Col-0 x Ler embryo sample, but not in the 6× washed Ler x Col-0 embryo sample, likely due to lower cDNA quality of this particular library. Nevertheless, Sanger sequencing analysis confirmed that those four MEGs show monoallelic expression in the 6× washed Col-0 x Ler sample (Figure S6A) and were thus classified as partially confirmed MEGs. Only one candidate gene that seemed to be imprinted in the 1× washed libraries could not be detected anymore in both 6× washed embryo samples, suggesting that this transcript is either lowly abundant in the embryo and/or was degraded during the extensive 6× washing procedure (*AT4G11960*, Figure S6C).

In conclusion, sequencing analysis of extensively washed, reciprocal 2–4 cell embryonic cDNA libraries confirmed seven MEGs and partially confirmed four MEGs as imprinted genes in the embryo, strongly suggesting that their monoallelic expression is not caused by seed coat or endosperm contamination.

### MEG and PEG Reporter Lines Show Imprinted Expression in the Embryo

In order to demonstrate parent-of-origin-dependent, monoallelic expression in the embryo using an independent assay, we cloned the promoter of seven MEGs (*AT1G29660, AT1G72260, AT2G47115, AT5G62210, AT3G20520, AT2G17710, AT3G21500*) and the single identified PEG (*AT3G26790*) as transcriptional fusions with the bacterial *uidA* reporter gene encoding ß-glucuronidase (GUS; [40]). We screened 24 independent T1 lines for all 8 constructs for expression in the seed. Except for the PEG reporter *pFUS3::GUS*, all MEG reporters exhibited fairly strong staining in the seed coat, making embryo expression analyses on whole seeds impossible (Figure S7). To assess whether the gene-of-interest is indeed expressed in the embryo as indicated by our previous analyses, we isolated self-fertilized embryos at early and late stages of two to three independent lines for each MEG construct in the T1 generation and stained them for GUS activity on slides. Whereas 6 lines showed strong expression in the embryo (Figure S8), one line (*pAT3G21500::GUS*) showed only very weak and hardly detectable GUS staining in the embryo (Figure S9) and was, therefore, not used for further analyses.

**Figure 3:**
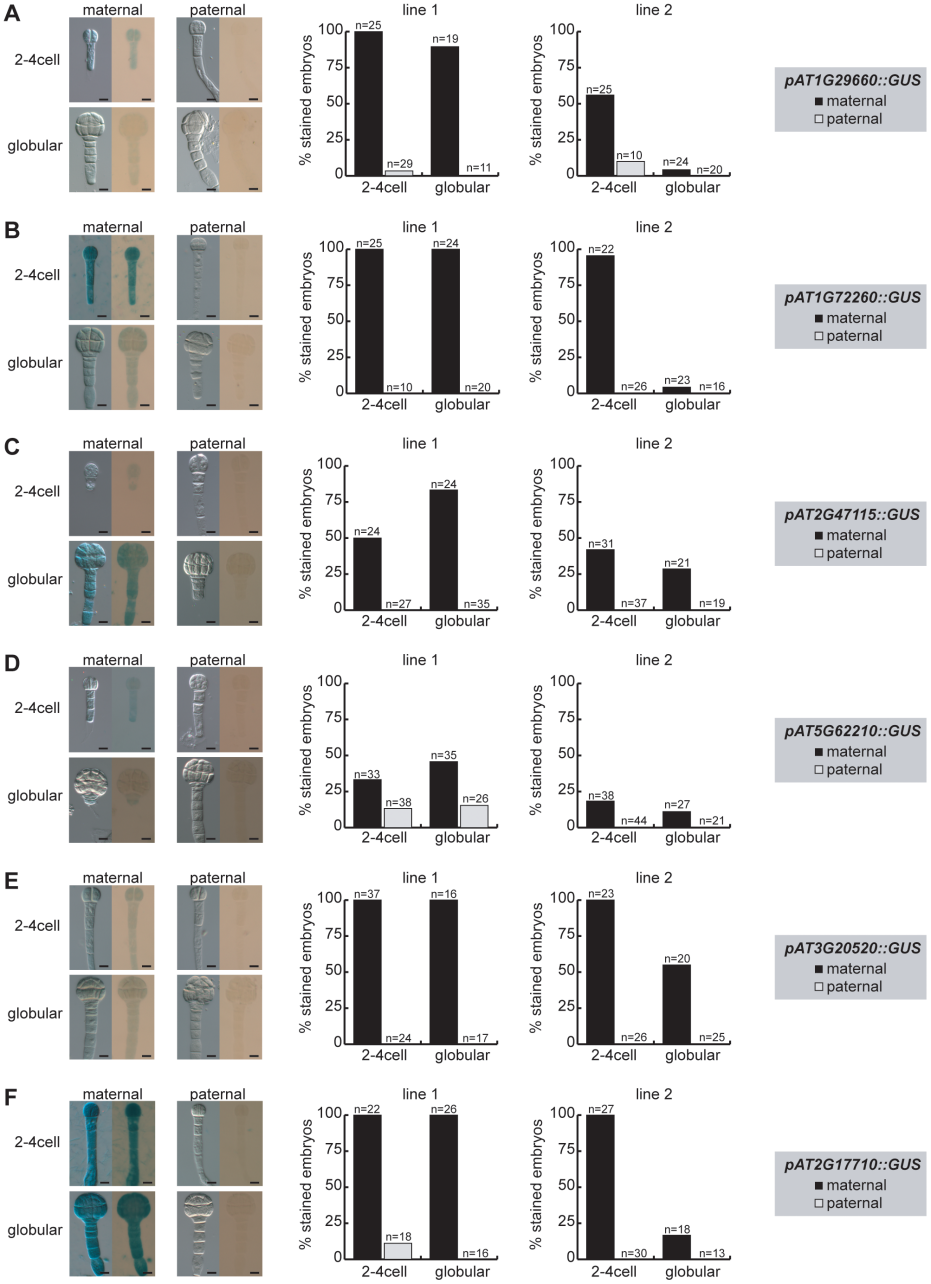
Parent-of-origin-dependent expression of MEG reporter lines in isolated embryos. The MEG reporter lines were reciprocally crossed to wild-type Col-0 plants and embryos were isolated at 2.5 DAP (2–4 cell embryos) and at 4 DAP (globular embryos) prior to GUS staining. Embryos were stained on slides for 4 days at 37°C and then analyzed for GUS expression using bright-field microscopy. For each line, two independent transgene insertions (line 1, line 2) were analyzed and quantified for maternal (black columns, maternally inherited reporter gene) and paternal expression (grey columns, paternally inherited reporter gene) and are displayed separately (middle panel and right panel). Embryo pictures of line 1 are shown on the left, always showing a DIC picture and a bright-field picture of each stage and direction of cross. The embryonic stages, maternal or paternal GUS reporter expression and the analyzed reporter line are indicated and the numbers of the quantified embryos are shown above each column. Scale bar = 10 µm. (A) p*AT1G29660::GUS*. (B) *pAT1G72260::GUS*. (C) *pAT2G47115::GUS*. (D) *pAT5G62210::GUS*. (E) *pAT3G20520::GUS*. (F) *pAT2G17710::GUS*.

To assess whether the promoter-GUS reporters are imprinted in the embryo, we performed reciprocal crosses between two independent reporter lines of each of the six strong MEG constructs (T2 generation) with wild-type plants (Col-0). F1 embryos were isolated at 2.5 DAP (∼2–4-cell embryos) and 4 DAP (∼globular embryos), and stained on slides for 4 days before analyzing them for GUS expression. For the MEG reporters, we expected to see the GUS signal only in the embryos that inherited the reporter gene maternally and not in the embryos that received the reporter gene from the pollen donor. We found that three of the six GUS-reporter lines are fully imprinted showing exclusive maternal expression (*pAT1G72260::GUS, pAT2G47115::GUS, pAT3G20520::GUS*, Figure 3B, 3C, and 3E), while the remaining three GUS-reporter lines show a very strong bias towards maternal expression (*pAT1G29660::GUS, pAT5G62210::GUS, pAT2G17710::GUS*, Figure 3A, 3D, and 3F). The PEG reporter line *pFUS3::GUS* shows very strong and embryo-specific expression starting from the 8-cell embryo stage both in whole-mount seed staining assays (Figure S7H) and after embryo isolation (Figure S10C). Since GUS expression for this gene seems to be very specific to the embryo (Figure S7H), no embryo isolation was necessary after reciprocal crosses to assess whether *pFUS3::GUS* shows imprinted expression. Yet, in contrast to the MEG reporter lines, we detected embryonic GUS expression no matter from which parent the reporter was inherited (Figure S10A and S10B). This suggests that the upstream regulatory region of *FUS3* is not sufficient to confer imprinted paternal expression in early *Arabidopsis* embryos. However, *pFUS3::GUS* activity was first detected at 3 DAP corresponding to the (4-)8 cell stage (Figure S10A). Thus, the level of gene expression at earlier stages, where we actually detected exclusively paternal expression using allele-specific expression analysis (Figure 1G), might be below detection level in this assay.

In conclusion, all the MEG and PEG reporter lines cloned and analyzed are expressed in the embryo (Figure 3, Figure S8, Figure S10). Moreover, all MEG reporter lines are either fully imprinted or show a strong bias for maternal expression (Figure 3). The upstream regulatory sequences that were cloned are, thus, sufficient to confer imprinted expression during early stages of embryogenesis. On the other hand, a few loci, such as *FUS3*, might require additional regulatory elements for imprinting. Finally, while all MEG reporter lines are expressed in the seed coat (Figure S7) in addition to the embryo (Figure S8), they are clearly regulated by genomic imprinting in the embryo itself (Figure 3).

### PRC2 but Not MET1 Is Involved in Regulating Genomic Imprinting in the *Arabidopsis* Embryo

In order to investigate how genomic imprinting is regulated in the embryo, we crossed mutants affecting imprinting regulators to wild-type parents of a distinct accession. DNA-methylation and histone modification, in particular H3K27me3 mediated by the PRC2, have both been shown to regulate genomic imprinting (for review see [10]). Therefore, we crossed the *fertilization-independent endosperm* (*fie*) mutant, in which PRC2-mediated repression is fully abolished, reciprocally to wild-type plants, and used the *met1-3* mutant, disrupting the maintenance of DNA-methylation in the CG-context, as a male donor to pollinate wild-type plants. *MET1* was thus far only implicated as a paternal repressor of imprinted loci, whereas PRC2 contributes to the regulation of MEGs (e.g. *MEA*) and PEGs (e.g. *PHE1*) (for review see [10]). Therefore, we crossed *fie* mutants reciprocally but the *met1-3* mutant only as a pollen donor. As before, we isolated embryos from the resulting F1 hybrid seeds and proceeded with RNA extraction and library amplification, creating mutant embryonic cDNA libraries (i.e. *fie*/*FIE* x Ler, Ler x *fie*/*FIE*, and Ler x *met1-3*/*MET1*).

**Figure 4:**
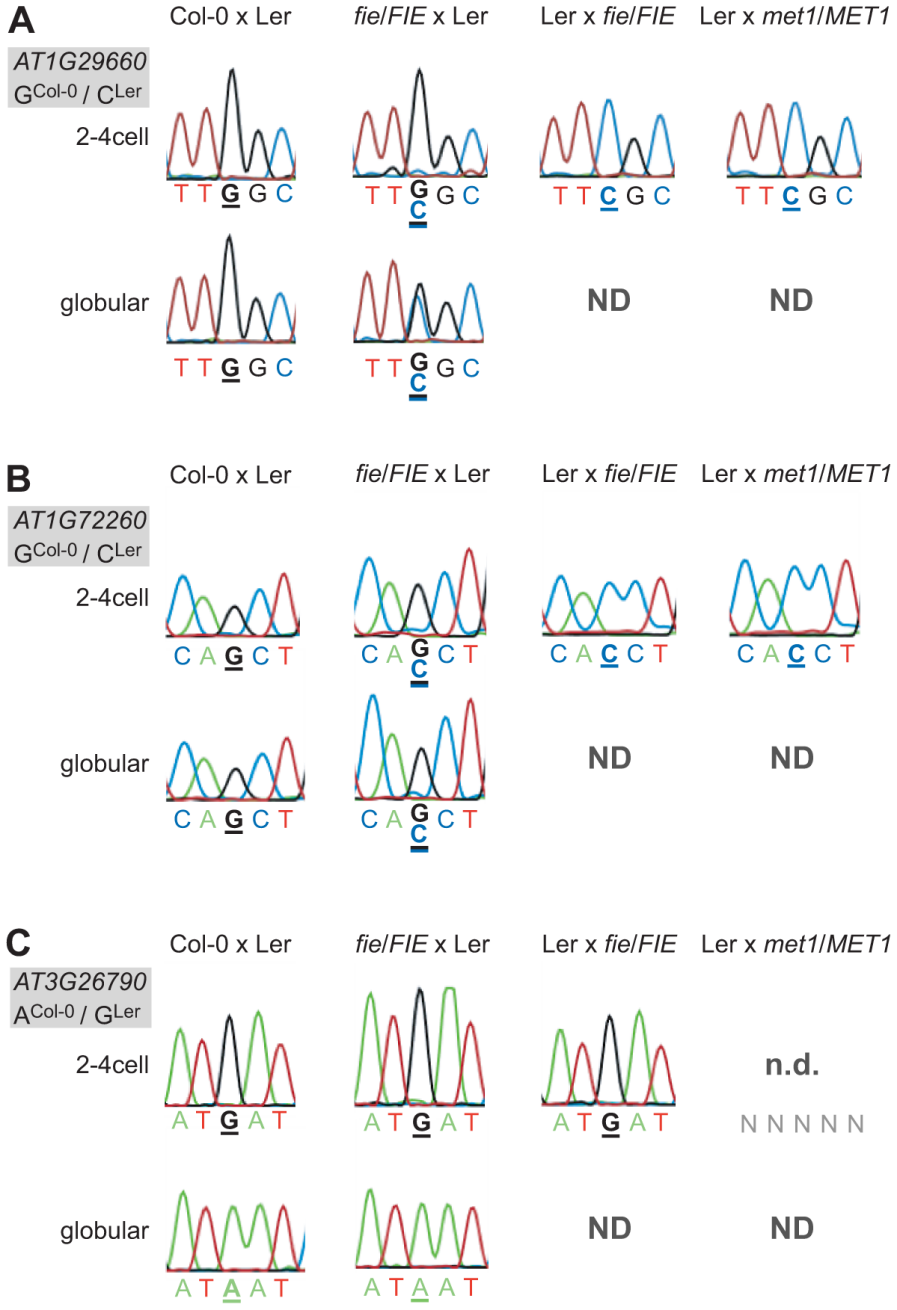
Effect of PRC2 and *MET1* function on imprinted expression in the embryo. Mutant embryonic samples were generated and the confirmed MEGs and the PEG were analyzed for derepression of the silent allele. Heterozygous *fie* mutants (in Col-0) were crossed maternally and paternally and heterozygous *met1-3* mutants (in Col-0) were crossed paternally to wild-type Ler as indicated above the chromatograms. To simplify the reading of the graph, we show again the wild-type situation of one replicate from the Col-0 x Ler cross in Figure 1. Embryos were isolated at 2.5 DAP (2–4 cell embryos) and at 4 DAP (globular embryos, only for the cross *fie*/*FIE* x Ler). The embryonic stage is indicated on the left, the analyzed gene and the polymorphism between the mutant (all in Col-0 background) and the wild-type allele (Ler) is shown in the grey box beside each panel. Furthermore, the polymorphic nucleotide is displayed in bold and underlined below each chromatogram. ND, not determined: indicates that the sample was not available; n.d., not detected: indicates that the transcript was not detected. (A) *AT1G29660*. (B) *AT1G72260*. (C) *FUS3* (*AT3G26790*).

We tested the allele-specific expression pattern of the eleven MEGs and the PEG in the Ler x *met1-3*/*MET1* mutant library (2–4cell stage) and found that all MEGs were still monoallelically expressed (Figure 4 and Figure S11). Thus, in contrast to some of the well-studied maternally expressed imprinted loci in the *Arabidopsis* endosperm (i.e. *FIS2*, *FWA*), disrupting paternal DNA-methylation maintenance does not appear to affect the imprinted expression of the embryonic MEGs at all (Figure 4A and 4B and Figure S11). Yet, the PEG could not be detected anymore in the Ler x *met1-3*/*MET1* mutant cDNA library, indicating a potential involvement of paternal *MET1* in activating the paternal *FUS3* allele (Figure 4C). Since half of the embryos are expected to inherit an unaffected *FUS3* allele from wild-type *MET1* pollen, the remaining *FUS3* transcript level may just be below our detection limit. This is in agreement with our earlier findings that, even in wild-type embryos, we are at the limit of detection for *FUS3* using this assay (Figure 1).

Disrupting PRC2 function by crossing *fie* mutants maternally or paternally did have an effect on two MEGs and on the PEG. A maternal *fie* mutation was able to derepress the paternal alleles of *AT1G29660* and *AT1G72260* (Figure 4A and 4B). The alleles were slightly derepressed at the 2–4 cell stage in both cases, but were differently affected by the *fie* mutation at the globular stage. While *AT1G29660* was fully derepressed and biallelically expressed (Figure 4A), the paternal allele of *AT1G72260* retained a low expression level (Figure 4B). While only one replicate was tested for each cross, the data were fully consistent for the two stages we analyzed. Interestingly, a paternal *fie* mutation induced maternal expression of the PEG *FUS3*, while abolishing its paternal expression (Ler x *fie*/*FIE*, 2–4cell stage, Figure 4C). These results indicate that paternal *FIE* activity is required – likely indirectly – to activate the paternal *FUS3* allele and that paternal FUS3 may negatively control the maternal *FUS3* allele after fertilization. Interestingly, the *FUS3* promoter contains two RY motifs, 484 bp and 1577 bp upstream of the start codon, respectively. FUS3, a B3-domain transcription factor, directly binds to the RY motif, which is present in many seed specific promoters [41]. This further supports the hypothesis that FUS3 autoregulates itself.

In conclusion, the PRC2 seems to be involved in regulating genomic imprinting in the embryo. Maternal PRC2 activity maintains the repression of the silent paternal allele of two MEGs after fertilization, while paternal PRC2 function together with paternal MET1 is somehow implicated in the activation of the paternal allele of the PEG *FUS3*. In contrast, paternal MET1 activity does not seem to play a role in regulating genomic imprinting of the eleven MEGs in the embryo. Our result indicates that there must be additional, so far unknown factors regulating genomic imprinting in the embryo.

### Disrupting Embryonic MEGs or the PEG Has No Effect on Seed Viability or Early Embryogenesis

A few of the genes imprinted in the endosperm, such as *MEA* and *FIS2*, show parent-of-origin-dependent seed abortion when mutated [42], [43]. To reveal a potential role of the confirmed embryonic MEGs and the PEG during embryogenesis and seed development, we analyzed T-DNA insertions if available (Table S2). We assessed seed viability by dissecting siliques and analyzing seed set of 16 to 24 individuals of a genotyped segregating population. None of the analyzed T-DNA insertion lines showed reduced seed set (Table S2). This suggests that the MEGs and the PEG we identified play only a subtle role during embryogenesis or do not show an effect on seed development due to redundancy or an incomplete disruption of the corresponding gene. Furthermore, we tried to assess more subtle effects by dissecting and clearing seeds of heterozygous mutant individuals, followed by morphological analysis of early embryogenesis. However, we could not observe any obvious patterning defects or other developmental aberrations in the lines analyzed (Table S2). Interestingly, homozygous *fus3* embryos show a phenotype late in seed development, namely a prolonged cell division phase in the embryo throughout seed maturation [44]. Yet, the late occurrence of this phenotype is unlikely to be caused by the early imprinted state of *FUS3*, and the zygotic recessive nature of the phenotype fits well with our observation that *FUS3* has a biallelic expression late in embryogenesis.

Taken together, we did not identify any obvious fertility or embryo patterning phenotypes when analyzing the available T-DNA insertion lines disrupting the imprinted embryonic genes we identified.

### Most MEGs and the PEG Are Contributed in a Parent-of-Origin-Dependent Manner in Different Ecotypes and at Later Stages

To determine whether our embryonic MEGs and the PEG are (i) indeed expressed in other embryonic samples, and (ii) monoallelically contributed in other *Arabidopsis* accessions and at different embryonic stages, we compared our data with the results of three recent studies. Xiang and colleagues isolated embryos from Col-0 wild-type plants manually, from zygote up to mature embryos, and performed transcriptome analysis using microarrays [45]. Eight of our MEGs and the PEG are expressed clearly above their background level at the 4-cell embryo and the globular embryo stage, whereas three MEGs are just at background level, which is considered not or lowly expressed (Table 1, Table S3). In contrast, these three MEGs are clearly expressed in young hybrid embryos from reciprocal crosses between the accession Col-0 and Cape Verde Islands (Cvi; [34]; Table 1, Table S4). This finding suggests that either the microarray technique is not sensitive enough to detect low expression levels, or that these three MEGs are stronger expressed in hybrids than in self-fertilized embryos.

**Table 1:** Embryonic expression and parent-of-origin expression of the confirmed MEGs and the PEG in other studies.

	Embryo expression^1^		Parent-of-origin of transcripts	
Gene	4 cell	globular	1–32 cell^2^	torpedo^3^
*AT1G29660*	YES	YES	biallelic	Ler bias
*AT1G72260*	YES	YES	maternal	maternal
*AT2G47115*	NO	NO	maternal	not expressed
*AT5G62210*	YES	YES	maternal	biallelic
*AT3G20520*	NO	NO	maternal	maternal bias
*AT2G17710*	YES	YES	biallelic	maternal bias
*AT3G21500*	YES	YES	maternal	maternal
*AT2G01520*	YES	YES	maternal bias	maternal
*AT1G20680*	NO	NO	maternal	maternal
*AT5G51950*	YES	YES	maternal bias	maternal
*AT1G29050*	YES	YES	no SNPs covered	maternal bias
*AT3G26790*	YES	YES	early PEG	biallelic

1 : The embryonic expression of the 11 MEGs and the PEG assayed by Xiang and colleagues [45] at the 4 cell and the globular stage are displayed. Detailed expression scores can be found in Table S3.

2 : The parent-of-origin-dependent expression of the 11 MEGs and the PEG in early embryos (1–32cell) of reciprocal crosses between Col-0 and Cvi [34] is shown. Detailed read counts can be found in Table S4.

3 : The parent-of-origin-dependent expression of the 11 MEGs and the PEG in late embryos (torpedo stage) of reciprocal crosses between Col-0 and Ler [28] is shown. Detailed read counts can be found in Table S4.

Furthermore, we compared parent-of-origin-dependent expression of our embryonic MEGs and the PEG to the parent-of-origin-dependent expression in (i) early Col-0 x Cvi embryos (different accession, similar stage; [34]), and (ii) late torpedo-stage Col-0 x Ler embryos (same accessions, but later stage; [28]). Interestingly, eight MEGs and the PEG show a monoallelic or at least a clearly biased parent-of-origin-dependent expression in early Col-0 x Cvi embryos confirming the results of our study (Table 1, Table S4). This suggests that genomic imprinting of those loci is conserved between the Col-0, Ler, and Cvi accessions. However, analyses of additional accessions would be required to clearly demonstrate locus- *versus* allele-specific imprinting at these loci. Two MEGs are biallelically expressed and one is low expressed and no SNPs were covered by reads, making a parent-of-origin analysis impossible (Table 1, Table S4). Yet, when looking at the same accessions later in development (torpedo stage), we found that eight MEGs are still expressed maternally or with a maternal bias (albeit most were covered by few reads only). One MEG shows a Ler bias, one MEG and *FUS3* are biallelically expressed, and one MEG is not expressed anymore (Table 1, Table S4). This indicates that at some loci imprinted expression is lost already during embryogenesis, whereas at other loci this happens later during or even after embryogenesis. This analysis shows that the majority of the analyzed loci that are imprinted in the embryo is also imprinted in different accessions [34] and maintains a parent-of-origin-dependent expression later during embryo development [28].

### Embryonic Imprints Are Erased or Ineffective in the Seedling

**Figure 5:**
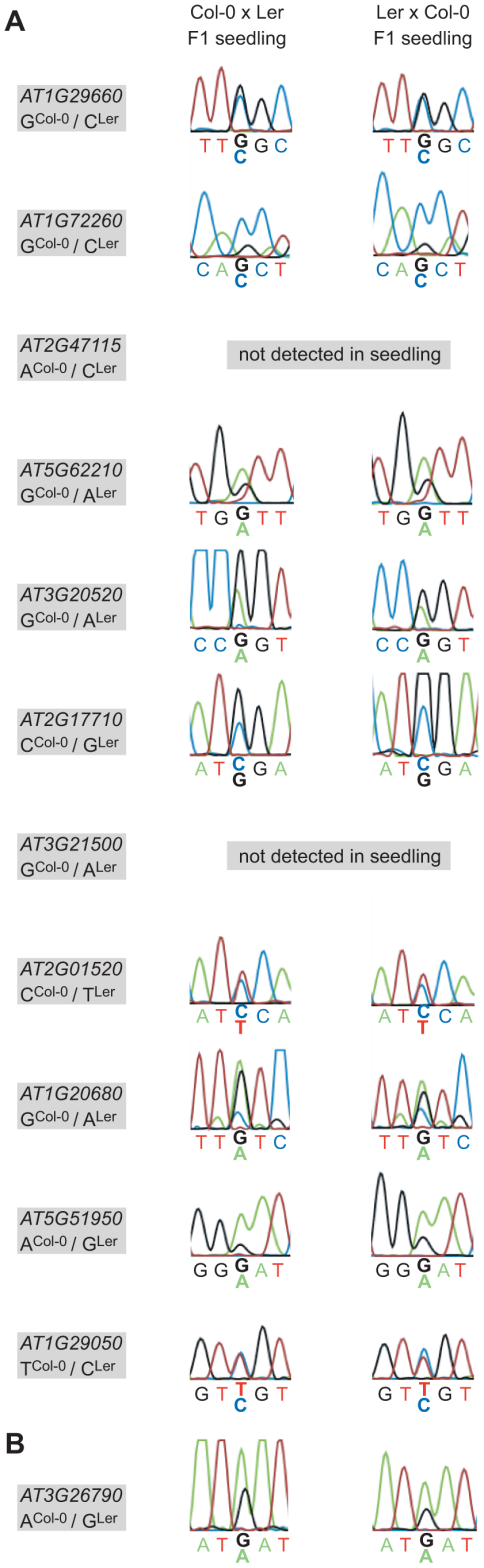
Allele-specific expression analysis of confirmed MEGs and the PEG in hybrid F1 seedlings. The allele-specific expression of the eleven confirmed MEGs (A) and the confirmed PEG (B) was assessed in reciprocal F1 hybrid seedling cDNA libraries (8 days after sowing). Nine MEGs and the PEG show biallelic expression in the seedling and two MEGs were not detected in the seedling samples, as indicated (*AT2G47115*, *AT3G21500*). The analyzed gene and the polymorphism between Col-0 and Ler are specified in the grey box beside each panel, whereas the direction of the cross is indicated on top.

Parent-of-origin-dependent expression seems to be maintained until late stages of embryogenesis, at least for some of the loci analyzed [28]. Eventually the imprint has to be erased, the very latest during gametogenesis. To address this question we reciprocally crossed Col-0 and Ler, grew F1 seedlings up to the 4-leaf stage (8 days after sowing), and produced hybrid F1 seedling cDNA libraries. We then performed allele-specific expression analysis using the MEG and PEG assays. 9 of 11 MEGs and *FUS3* could be amplified from the seedling library and are thus expressed in the seedling. The two genes that were not amplified (*AT2G47115*, *AT3G21500*) are either not expressed in the seedling or below the detection level of our assays. Sanger sequencing revealed that all MEGs and *FUS3* are expressed from both parents in F1 hybrid seedlings (Figure 5). This suggests that the imprint is erased late during embryogenesis or early during vegetative development, but long before flowering and the initiation of reproductive development. Alternatively, the imprint might persist in the seedling but is ignored by the transcriptional machinery, leading to the observed biallelic expression pattern. For instance, some loci may be transcribed from an imprinted promoter in the embryo and from an alternative, non-imprinted promoter in the seedling, or the imprinting control elements may not be accessible any more to the corresponding *trans*-acting factors in seedlings.

## Discussion

### Parent-of-Origin-Dependent, Monoallelic Gene Expression Is Not Restricted to the Endosperm in *Arabidopsis*

Recently, two studies independently identified monoallelically-derived transcripts in early *Arabidopsis* embryos when assessing the genome-wide parental contribution to plant embryogenesis [33], [34]. Analysis of reciprocal Col-0 x Cvi F1 embryos identified more than 100 potentially imprinted or maternally/paternally deposited transcripts in the *Arabidopsis* embryo [34], whereas we identified 50 potential MEGs and 30 potential PEGs by assessing the allele-specific transcriptome of Ler x Col-0 embryos [33]. We focused on 18 MEG and six PEG candidates that showed strong expression in the embryo but no expression in the gametes. We could confirm eleven MEGs expressed at the 2–4 cell and globular embryo stage and one PEG at the 2–4 cell stage using RT-PCR and Sanger sequencing on replicated and reciprocal hybrid, embryonic cDNA samples (Table 2, Figure 1, Figure S4). In addition, reporter gene analysis of seven MEGs and one PEG independently confirmed embryonic expression and six MEG reporter lines were fully imprinted or expressed with a strong maternal bias in the embryo (Table 2, Figure 3, Figure S8, Figure S9). Furthermore, absence of most MEG or PEG transcripts in the gametes strongly suggests *de novo* expression of the genes in the embryo, although the gametic trancriptome was only assessed by microarrays, which are less sensitive than RNA-Seq (Table 2, Table S1, [35], [36], [46]. However, the fact that most of those genes were found expressed in a microarray study of embryogenesis – while being absent in gametes - supports their *de novo* expression after fertilization ([45]; Table 1, Table S3). The MEGs were not only expressed in the embryo, but also in the surrounding seed coat (Figure S7). Therefore, examining potential maternal sporophytic contamination was essential to our study. To do so, we produced two extensively washed (6×), reciprocal 2–4 cell embryonic cDNA libraries that should be completely devoid of all potential contamination. Allele-specific expression analysis in those samples revealed pure monoallelic expression of the 11 MEGs and the PEG in the embryo, affirming their imprinted expression patterns (Figure 2, Figure S6). In addition, we isolated embryos derived from reciprocal crosses between MEG reporter lines and wild-type plants prior to GUS staining. Thus, the observed GUS signal is embryo-specific and cannot be due to diffusion from the seed coat.

**Table 2:** Overview of the embryonic MEGs and the embryonic PEG.

GENE	GO-term		Monoallelic?	GUS in embryo?	in gametes?	regulation
*AT1G29660*	lipid metabolism	MEG	YES	YES^3^	NO	PRC2
*AT1G72260*	defense (JA)	MEG	YES	YES^3^	YES	PRC2
*AT2G47115*	unknown	MEG	YES	YES^3^	not on array	?
*AT5G62210*	lipid metabolism	MEG	YES	YES^3^	NO	?
*AT3G20520*	lipid metabolism	MEG	YES	YES^3^	NO	?
*AT2G17710*	unknown	MEG	YES	YES^3^	NO	?
*AT3G21500*	terpenoid metabolism	MEG	YES	YES^4^	NO	?
*AT2G01520*	meristem phase transition	MEG	YES^1^	ND^5^	NO	?
*AT1G20680*	unknown	MEG	YES^1^	ND^5^	NO	?
*AT5G51950*	redox function	MEG	YES^1^	ND^5^	NO	?
*AT1G29050*	unknown	MEG	YES^1^	ND^5^	NO	?
*AT3G26790*	embryogenesis	PEG	YES^2^	YES^6^	NO	PRC2

1 : Monoallelic expression is only confirmed in 6× washed Col-0 x Ler control sample.

2 : Monoallelic expression of *FUS3* (*AT3G26790*) only in early embryos (2–4 cell).

3 : Reporter is expressed and imprinted in the embryo.

4 : Reporter is weakly expressed in the embryo and imprinted expression was not assessed.

5 : ND, not determined: indicates that the reporter line is not available.

6 : Reporter is expressed in the embryo but not imprinted.

Apart from the controversially discussed *MEA* and *PHE1* genes [11], the maize *mee1* gene is clearly imprinted in plant embryos [7]. Other recently published studies performed genome-wide analysis of parent-of-origin allelic expression in endosperm and embryos. Hsieh and colleagues identified 37 MEGs and one PEG in the *Arabidopsis* embryo 7–8 DAP, but discarded them because of the possibility that these data may be due to contamination by endosperm and/or maternal tissues [27]. Gehring and coworkers found 17 MEGs and one PEG in *Arabidopsis* embryos 6 DAP (torpedo stage) but none was further analyzed [28]. In rice, a similar study identified seven putative MEGs in the embryo and confirmed one, *Os10g05750*, using RT-PCR and Sanger sequencing, but its expression status in the gametes was not assessed [8]. Furthermore, Waters and colleagues identified 29 MEGs and 9 PEGs from a dataset of maize embryos 14 DAP, but did not follow up these findings with additional experiments [31]. In all studies, many of the embryonic MEGs and PEGs are also expressed and imprinted in the endosperm, which leads the authors to attribute their pattern of expression to endosperm contamination, and do not consider it a real, intrinsic and embryo-specific feature. Similarly, the confirmed eleven MEGs of this study are not only expressed in the embryo, but also in the seed coat, the seedling and/or the endosperm (Figure S7). *AT1G20680* even passed the statistical criteria used in two independent studies and is thus considered an imprinted gene in the endosperm [28], [29]. Many of our MEGs and the PEG are expressed in the endosperm and almost all MEGs are maternally expressed in the endosperm at 6 DAP (Table S5). Yet, allele-specific expression in stringently washed embryo samples and the analysis of reporter constructs strongly suggest that the embryonic parent-of-origin-dependent expression pattern is not due to contamination.

In contrast, we cannot fully exclude the possibility that mRNAs are transported from the endosperm to the embryo. To our knowledge mRNAs can only be transported symplastically via plasmodesmata [47]. However, this seems highly unlikely because the embryo is symplastically isolated from the endosperm during early seed development, due to the presence of cuticular material around the embryo ([48] and references therein). Even in the few cases where plasmodesmata between the suspensor and the endosperm have been described - only in the Fabaceae and Crassulaceae families - they are associated with electron-dense material, probably blocking the plasmodesmata [48]. Taken together, we expect that a careful reexamination of the embryonic MEG and PEG candidates found in other studies could confirm additional genes regulated by genomic imprinting in the embryo, as it is the case for the genes reported in this study.

Comparison of this study's 80 imprinted candidates with all embryonic MEG and PEG candidates identified in different studies reveals no genes to be common in all studies and only three overlapping genes between two studies (*AT2G01520*, *AT1G49450* and *AT1G57800*; [27], [28], [34]). Similarly, the overlap between four recent studies [27]–[30] identifying imprinted genes in the endosperm of *Arabidopsis* is minor, with only 20 genes of more than 300 being shared between two studies analyzing the same ecotypes and similar developmental stage [49]. The low number of common genes may be explained, at least to some extent, by the use of different accessions and the analysis of different developmental stages. Thus, some of the potential MEGs and PEGs, in both the embryo and endosperm, seem to be imprinted allele-specifically rather than locus-specifically and/or imprinting could be specific to a given developmental stage. In addition, different statistical procedures to select potentially imprinted genes may also contribute to the discrepancies [50]. Analyzing the datasets prior to stringent filtering increases the overlap [29], as well as treating two different datasets with the same statistical pipeline [28]. Taken together, the recent rapid development of high-throughput transcriptome sequencing allows large-scale identification of imprinted genes in different organisms and tissues. Yet, the obvious discrepancy between different datasets shows that in-depth analyses and substantial validation of imprinted candidate genes using alternative methods is necessary.

In conclusion, we show that at least nine MEGs and one PEG are regulated by genomic imprinting in the embryo and, in addition, two MEGs display steady-state monoallelic expression in the embryo. Likely, the list of MEGs and PEGs in plant embryos will expand in the future by validating additional candidates or by high-throughput sequencing of additional embryonic samples.

### Delayed Paternal Gene Activation in the Embryo Is Distinct from Embryonic Imprinting

In a former study, we have shown that the embryo transcriptome of intraspecific (Ler x Col-0) hybrids showed an overall maternal dominance, albeit 66% of the genes were biparentally expressed [33], [51]. Although this wide-spread maternal dominance was recently challenged by a study using different accessions and amplification techniques [34], [51], our genetic analyses further demonstrated that the underrepresentation of paternal transcripts is regulated by a maternal siRNA-based silencing mechanism [33]. However, DNA methylation and PRC2-mediated repression, two epigenetic pathways often associated with imprinting, were not involved [33]. In addition, the vast majority of genes that were maternally dominant at the 2–4 cell stage have a higher paternal contribution at the globular stage. The biallelic control gene used in this study, *AT1G02780*, is a good example for a gene showing biased expression in 2–4 cell embryos but an equal parental contribution at the globular stage, a pattern that we confirmed using allele-specific expression analysis (Figure S1A, [33]).

In contrast, for all potentially imprinted genes identified in this study exclusively reads from one parent only were found in all stages and replicates (Table S1). In addition, for the 11 confirmed embryonic MEGs and the PEG we never detected any paternal or maternal SNP signatures, respectively, in the chromatograms of all samples and stages analyzed. This strongly suggests that they are indeed monoallelically expressed and not only maternally biased transcripts, as it is the case for the biallelic control gene that shows a low but clearly visible paternal SNP signature already at the 2–4 cell stage (Figure S1A). Similarly, three of six MEG reporter lines were completely silent if paternally inherited, whereas another three showed paternal expression but at a very low frequency at both stages analyzed, suggesting an imprinted expression pattern in the embryo (Figure 3). In contrast, the GUS marker lines analyzed by Autran and colleagues showed a much higher frequency of paternal expression already in 2–4 cell embryos and most were fully active by the globular stage [33].

In conclusion, delayed paternal genome activation is a genome-wide, gradual process leading to a maternal bias during early embryogenesis. In contrast, the embryonically imprinted genes described in this study are completely monoallelically expressed at both the 2–4 cell and globular stage of embryogenesis. Of course, maternally expressed imprinted loci will contribute to the maternal dominance in the transcriptome of early embryos [33]. However, maternally biased expression can also be due to the deposition of maternal transcripts produced prior to fertilization, and we currently do not know what fraction of these loci are *de novo* transcribed in a parent-of-origin-dependent way after fertilization, i.e. are regulated by imprinting [52].

### PRC2 but Not MET1 Is Involved in Maintaining Repression at Silent Alleles

The regulation of monoallelic and parent-of-origin-dependent gene expression largely depends on differential DNA methylation of the parental alleles in mammals and of some imprinted loci in the plant endosperm [10]. We analyzed the effect of a paternal *met1-3* mutation, known to derepress silent paternal alleles of *FWA* and *FIS2* [16], [18] in the endosperm, on imprinted gene expression in the embryo. We did not find any effect of paternal *met1-3* on the expression of the 11 MEGs, suggesting that MET1-mediated DNA-methylation in the CG context is not important for the regulation of embryonic MEGs (Figure 4, Figure S11). By contrast, *met1-3* abrogated paternal *FUS3* expression, indicating a role of *MET1* in activating paternal *FUS3* (Figure 4C). Similarly, expression of the paternal *PHE1* allele is reduced if inherited by a *met1* mutant pollen [53]. However, further studies are required to confirm this observation and to rule out that *FUS3* is not just below detection level in this sample, as it was the case in two wild-type samples we analyzed (Figure 1G).

The second, well-established imprinting regulator is PRC2, which mediates H3K27me3 [10]. In fact, we found that in embryos lacking maternal *FIE* activity, the usually silent paternal alleles of two MEGs were derepressed (Figure 4A and 4B). Similarly, the imprinted genes *MEA* and *AtFH5* are biallelically expressed in seeds with a maternal mutation in a PRC2 subunit [17], [19], [26]. In addition, the paternal *MEA* allele was shown to be partially derepressed by a paternal mutation in PRC2 components, which is not the case for the identified MEGs in the embryo [26]. However, this suggests that maternal PRC2 is involved in maintaining the silent state of paternal alleles of imprinted genes in both, the endosperm and the embryo. In addition, in embryos inheriting a paternal *fie* mutation, the expression pattern of the PEG was inverted: Instead of being paternally expressed, *FUS3* seems to be solely maternally expressed (Figure 4C). Our result suggests that paternal *FIE* is somehow required to activate the paternal *FUS3* allele, and that paternal *FUS3* is involved in the repression of the maternal *FUS3* allele just after fertilization. Interestingly, the presence of RY motifs in the *FUS3* promoter suggests that FUS3 can indeed bind to its own promoter. Such a negative feedback regulation of the gene product on its own imprinted expression has also been described for *MEA* [17], [24], [26]. However, whereas paternal PRC2 seems to be involved in the activation of the paternal *FUS3* allele, imprinted expression of *PHE1* requires maternal PRC2, which in this case is involved in the repression of the paternal allele of *PHE1* [9]. This indicates that the requirement of PRC2 for regulation of imprinted expression of PEGs differs between the two fertilization products at least for these two loci.

We could not find any effect of mutations in *MET1* and *FIE* on 9 MEGs. Thus, other mechanisms must be involved in regulating parent-of-origin-dependent gene expression in the embryo. As proposed for the endosperm [29], asymmetric, non-CG DNA methylation could be involved in silencing the paternal alleles. However, asymmetric DNA methylation in the CHG context involves the SUVH4 methyltransferase *KYP* [54] and all of the identified MEGs and PEGs are still imprinted in the mutant *kyp* x Ler embryonic library ([33]; Table S1). Thus, asymmetric DNA methylation in the CHG context seems unlikely to play a role in regulating the remaining 9 MEGs. Any other epigenetic mark could account for, or contribute to, the imprinted expression pattern in the embryo. Especially histone modifications might be of importance since they are more readily reversible than DNA methylation [55] and different modifications have been associated with imprinted genes in maize [56].

### Erasing and Resetting the Imprint

In mammals, imprinting marks are erased and reset during germ line development. Very early during embryogenesis, as the germ line is set aside, epigenetic marks are erased and re-established according to the embryos' sex [2], [3]. In plants, no germ line is set-aside, and the gametes develop very late from differentiated sporophytic cells. In the endosperm, one-way control of imprinting is sufficient, since the it does not contribute to the next generation. In contrast, imprints on embryonic MEGs and PEGs have to be erased and reset. The only well-studied, imprinted gene in the plant embryo is the maize gene *mee1* [7]. Interestingly, both alleles are fully methylated in the gametes and the maternal allele gets specifically demethylated in the zygote, indicating an additional, yet undiscovered primary imprinting mark. During embryogenesis the maternal allele continuously regains methylation and consequently becomes silent [7]. Thus, we do not know whether remethylation is cause or consequence or even involved in resetting the imprint, and we cannot speculate about the time of imprint erasure. In rice, the monoallelic expression pattern of *Os10g05750* is maintained throughout development in the endosperm, but in the embryo, *Os10g05750* starts to be expressed biallelically from 8 DAP. This suggests erasure of the potential imprint during late embryogenesis [8].

In *Arabidopsis*, we found that all MEGs and the PEG show biallelic (n = 10) or no (n = 2) expression in the early seedling; thus, the imprint must be erased – or become ineffective - either late in embryogenesis or very early during vegetative development. When analyzing the expression pattern of the 11 embryonic MEGs and the PEG in the allele-specific dataset of torpedo-staged embryos [28], we found that most genes are still expressed monoallelically or with a parental bias (n = 8), although at very low levels. Thus, complete erasure of the imprint seems to occur after the torpedo stage for most of the MEGs. Alternatively, the requirements for transcription of these genes might be different in seedlings and embryos, and the primary imprints might persist but do not control monoallelic expression any more later in development.

However, how an imprint is erased during late embryogenesis and reset during gametogenesis is unknown. In the case of the two MEGs where the repression of the maternal allele is maintained by PRC2 - likely by the seed-specific MEA-FIS2 complex [57] - it is tempting to speculate that, with decreasing expression of *MEA* during seed development [24], activity of the seed-specific PRC2 is decreasing and the H3K27me3 imprint might get lost by passive dilution during embryogenesis. However, a detailed analysis of epigenetic marks at embryonic MEG and PEG loci in gametes, embryos, and vegetative tissues would be required, which is extremely challenging due to the limited accessibility of gametic and embryonic tissue. In the future, advanced approaches will identify the primary imprinting mark(s) in the embryo, which will shed light on the yet unknown mechanism of erasing and resetting the imprints at embryonic MEGs and PEGs.

### Biological Significance and Evolution of Genomic Imprinting in the Embryo

In placental mammals and in flowering plants, mutations in many imprinted genes cause growth defects in embryo and/or the nourishing tissue (i.e. placenta or endosperm) in a parent-of-origin-specific manner [43], [58]–[64]. Growth defects are consistent with a role of genomic imprinting in a parental conflict over resource allocation from the mother to the developing offspring [65]. Yet, when we analyzed available T-DNA lines disrupting embryonic MEGs, we could not find fertility, patterning, or obvious growth phenotypes. This might be a result of gene redundancy and/or of a subtle role these genes play during embryogenesis and seed development, which is not revealed in controlled and non-competitive laboratory conditions.

Notably, 5 of 11 MEGs have a role in metabolism, whereas 4 others are of unknown function (Table 2). In addition, all MEGs are expressed in the maternal seed coat and some MEG reporter lines and the PEG reporter line show a slightly biased expression towards the basal embryo and the suspensor (Figure 3, Figure S8, Figure S10). This suggests that embryonic MEGs might have a function at the interface between embryo and mother, possibly by linking seed coat metabolism and embryo metabolism and rendering the genes in the embryo under maternal control. This would be in line with the co-adaptation imprinting hypothesis: It predicts maternal expression of genes affecting mechanisms that are crucial at the maternal-offspring interface [66], [67]. In addition, the co-adaptation hypothesis predicts that the number of MEGs must be much higher than the number of PEGs, at least in species where the offspring develops within the mother. In fact, we find a large excess of MEGs (>90%), reminiscent of all other studies that analyzed parent-of-origin allelic expression in plant embryos. Both studies analyzing *Arabidopsis* embryos call 97% or 94% MEGs, respectively [27], [28]. In rice, only embryonic MEGs were called [8], and in maize embryos 76% of the imprinted candidates in the embryo show maternal expression [31]. Also in the *Arabidopsis* and the rice endosperm more MEGs than PEGs were identified, but the fraction of embryonic MEGs is still higher than the fraction of endosperm-specific MEGs [27]–[30]. This suggests that the co-adaptation hypothesis might be of importance for the evolution of genomic imprinting in the embryo, whereas parental conflict might drive evolution in the nourishing tissue, the endosperm. Nevertheless, the evolution of genomic imprinting is likely due to a combination of parental conflict, mother-offspring co-adaptation, and other factors, depending on the locus and the tissue of expression.

In conclusion, we describe and confirm parent-of-origin-dependent, imprinted monoallelic expression in the *Arabidopsis* embryo. PRC2 is involved in the regulation of parent-of-origin allelic expression at some loci analyzed, but by far not all, suggesting additional, yet undiscovered regulators of genomic imprinting in the *Arabidopsis* embryo. Probably, the imprint is erased late in embryogenesis or early in vegetative development since all genes are either expressed from both parental alleles or not at all in young seedlings. However, what the primary imprint is and when and how exactly it is reset, is currently unknown. Future research will likely confirm some of the embryonic MEGs and PEGs from other studies and will help elucidating the regulation, erasure and resetting of genomic imprints in the embryo.

## Materials and Methods

### Plant Material and Growth Conditions

Columbia-0 (Col-0) and Landsberg *erecta* (Ler) are the standard wild-type accession used in this study. We reciprocally crossed Col-0 and Ler to produce the hybrid embryonic samples and Col-0 was used for all *Agrobacterium*-mediated transformations in this study. The *fie*/*FIE* mutant (Col-0 background) used is SALK_042962 and the line has been described in detail in [68]. The *met1-3*/*MET1* mutant (Col-0 background) used was first described in [69], and was only propagated heterozygously. It was assessed for full methylation at the 180 bp CEN-repeat by Southern blot analysis before crossing, indicating an unaltered epigenetic landscape and excluding uncontrollable, indirect effects [20]. The *met1-3* genotyping assay is described in [20]. All plants were grown in a greenhouse chamber with 16 h light at ∼20°C and 8 h dark at ∼18°C with an average of 60% humidity. For crosses, plants were emasculated and pollinated 2 days later.

### Calling Potentially Imprinted Genes in the Embryo

The dataset from [33] was analyzed and we called all genes that had a q-value bigger than 0.8 (strong mono-parental bias, mi>0.8 and pi>0.8 for MEGs and PEGs, respectively) in all sequenced samples (2–4 cell Ler x Col-0, 2–4 cell *kyp*/*KYP* x Col-0, globular Ler x Col-0), in the 2–4 cell samples only, or in the globular wild-type sample only. All filtered genes were then compared to the second replicate run and only kept if they still showed reads from one parent only [33]. We also accepted genes that were sequenced in the globular sample of replicate 1 only (SOLiD 2009) and were not detected in the second replicate (SOLiD 2010). This procedure yielded 50 potential MEGs and 30 potential PEGs (Table S1). Expression levels (coverage by covered base; [33]) and present/absent calls in the egg cell and sperm cell [35], [36] were used to prioritize the potential embryonic MEGs and PEGs. MEGs and PEGs being highly expressed and showing preferably absent calls in the gametes (egg cell or sperm cells) were selected for in-depth analysis (i.e. RT-PCR and Sanger sequencing).

### Preparation of Hybrid Embryonic cDNA Libraries

Different wild-type accessions and/or mutant lines were reciprocally crossed as indicated in the main text, the figures, and figure legends to produce hybrid F1 seeds. For the 1×-washed wild-type embryonic samples we produced two independent biological replicates for each stage and direction of cross (i.e. 8 samples). The 2–4 cell embryos were isolated from seeds ∼2.5 days after pollination (DAP), whereas the globular embryo stage was isolated from seeds ∼4 DAP under our growth conditions. Embryo isolation was essentially performed as described in [33] with 5 additional washes after isolation for the extensively, 6× washed control samples (2–4 cell stage, reciprocally crossed). RNA was extracted using the Arcturus PicoPure RNA Isolation kit (Applied Biosystems) and the cDNA library amplified using the Ovation Pico WTA System (NuGEN) according to the manufacturer's protocol. As recommended by the Ovation Pico WTA System (NuGEN), we purified the cDNA libraries with the QIAquick PCR Purification Kit (QIAGEN) according to NuGEN's protocol. We used the Agilent 2100 Bioanalyzer (Agilent Technologies) to control cDNA library quality and measured quantity using Nanodrop. In addition, we controlled library quality and absence of genomic DNA contamination by RT-PCR amplifying *ACT11* and *WOX9*, an embryo-specific gene [70]. Furthermore, we tested the purity of the embryonic libraries by RT-PCR amplifying *TT10* and *AT5G42530*, two seed coat markers [37], and *FWA, AGL46*, and *AGL62*, three endosperm markers [16], [38], [39]. All primer sequences are specified in Table S6. The combined seed coat/endosperm sample consisted of apical pieces of seed coat and endosperm tissue after embryos were popped out and isolated. The cDNA library was produced like the embryonic libraries described above. In order to produce hybrid F1 seedling cDNA libraries and hybrid F1 seedling genomic DNA samples, we crossed Col-0 and Ler reciprocally, germinated the F1 hybrid seeds on plate and harvested them 8 days after sowing. Genomic DNA was extracted using the QiaQuick DNeasy kit (QIAGEN) and RNA was extracted using the NucleoSpin RNA Plant Kit (Machery-Nagel). Reverse transcription was performed as previously published [24].

### RT-PCR and Sanger Sequencing

RT-PCR was performed on diluted cDNA libraries (4 ng/µl) by doing 28 to 34 cycles (94°C for 15 sec, 58°C for 20 sec, and 72°C for 30 sec) followed by 72°C for 5 min. We used Sigma Taq DNA Polymerase and PCR buffer from Sigma-Aldrich and a final concentration of 2 mM MgCl_2_, 0.2 mM dNTPs and 0.2–0.4 mM Primer. The resulting PCR product was analyzed on a standard DNA agarose gel and the remaining product was purified using the NucleoSpin Gel and PCR Clean-up kit (Macherey-Nagel). The purified PCR product was Sanger sequenced and the chromatograms analyzed at the site of the SNP between Ler and Col-0 to assess its parent-of-origin. All assays were tested for non-biased amplification of sequence fragments from both accessions using genomic DNA of F1 hybrid seedlings (Col-0 x Ler and Ler x Col-0). The quantitative nature of the Sanger approach was tested using the same protocol, with 30 PCR cycles and 1.0 µl of the following mixes of Ler/Col-0 genomic DNA (20 ng/µl): 1∶9, 1∶3, 1∶1, 3∶1 and 9∶1 (Ler:Col-0). All sequences of the used primer are specified in Table S6.

### Reporter Lines: Cloning, Transformation, and Analysis

All GUS reporter lines were cloned using the pBGWFS7 vector (VIB, University of Gent), carrying a BASTA resistance gene (plant selection), a spectinomycin resistance gene (bacterial selection), and a Gateway-cloning cassette followed by eGFP and a *uidA* gene encoding ß-Glucuronidase (GUS) in frame. We amplified the upstream promoter region (from the previous gene until the start codon or a maximum of 2.5 kb of promoter sequence) of seven MEGs and one PEG containing the attB recombination sites in a two-step PCR reaction: First, we used chimeric primers comprising template-specific sequences plus the first 12 bases of the attB1 or attB2 sequence at the 5′-end. PCR was performed with the Phusion High-Fidelity DNA Polymerase (Finnzymes) and buffer, 0.2 mM dNTPs, 0.4 µM Primers using the attB adapter program 1 (98°C for 60 sec; 5 cycles of 98°C for 10 sec, 63°C for 20 sec, 72°C for 60–180 sec; 30 cycles of 98°C for 10 sec, 68°C for 20 sec, 72°C for 60–180 sec; 72°C for 300 sec). After analyzing the product on a gel, we used 1 µl of the 50× diluted first PCR product as template for the second PCR using attB adapter primers (attB1-adaptor: 5′-GGGGACAAGTTTGTACAAAAAAGCAGGCT-3′ and attB2-adaptor: 5′-GGGGACCACTTTGTACAAGAAAGCTGGGT-3′) to complete the attB sites. PCR was performed as above using the attB adapter program 2 (98°C for 60 sec; 5 cycles of 98°C for 10 sec, 48°C for 20 sec, 72°C for 60–180 sec; 15 cycles of 98°C for 10 sec, 58°C for 20 sec, 72°C for 60–180 sec; 72°C for 300 sec). The resulting PCR product containing the promoter sequence and the complete attB recombination sites were precipitated with polyethylene glycol, and the BP reaction (using pDONR221) and LR reactions were performed according to the manufacturer's recommendations (Invitrogen). The resulting expression vectors were transformed into competent *Agrobacterium tumefaciens* (GV3101), which were used to transform Col-0 plants by floral dipping [71].

### GUS Reporter Assays on Isolated Embryos

We first selected T1 lines strongly expressing the GUS reporter gene in the seed by staining young siliques overnight at 37°C after vacuum-infiltration (5–10 min) of the tissue in standard GUS staining solution (2 mM 5-bromo,4-chloro,3-indolyl-D-glucuronide (Biosynth-AG), 10 mM EDTA, 0.1% Triton X-100, 2 mM potassium ferrocyanide, 2 mM potassium ferricyanide, 50 mM phosphate buffer pH 7.2). Strongly expressing lines were selected, and reciprocally crossed with wild-type Col-0 plants. We then isolated embryos 2.5 DAP (2–4 cell stage) and 3.5 to 4 DAP (globular stage) in GUS staining solution (as above but with 0.5 mM potassium ferro- and ferricyanide instead of 2.0 mM for higher GUS activity). We directly transferred the isolated embryos on a microscope slide, added fresh GUS staining solution, covered the embryos with a coverslip, and stained them without vacuum-infiltration for 4 d at 37°C in plastic boxes with high humidity to prevent drying of the samples. After 4 days we analyzed the isolated embryos for GUS reporter expression using bright-field microscopy (Leica DMR) to ensure maximum sensitivity for GUS detection.

### Mutant Analysis

Available T-DNA insertion lines disrupting confirmed MEGs and PEGs (see Table S2) were ordered (2 lines/gene, if available). A mutant population (i.e. 24 individuals) was genotyped using primers flanking the insertion site (see Table S6, designed with the T-DNA primer design homepage http://signal.salk.edu/tdnaprimers.2.html) and the appropriate left border primer (for SALK lines: LBb1.3; for SAIL lines: Syg_LB1; for GABI lines: GBF_AC161_LB1; for FLAG lines: FL_LB4; for sequences see Table S6) using a standard PCR program (94°C for 15 sec, 58°C for 20 sec, and 72°C for 75 sec, 36 cycles). Then, mature siliques of each genotyped individual were opened to analyze the seed set. In addition, we harvested siliques at different developmental stages of one (usually heterozygous) mutant individual, dissected the seeds in modified Hoyer's solution (70% w/v chloralhydrate, 4% w/v glycerol, 5% w/v gum arabicum), and examined embryo patterning and development from the zygote to the torpedo stage using differential interference contrast (DIC) microscopy (Leica DMR).

## Supporting Information

Figure S1 Allele-specific expression analysis of the biallelically expressed control gene *AT1G02780*. (A) Reciprocal hybrid embryos were isolated at 2.5 DAP (2–4 cell embryos) and at 4 DAP (globular embryos) and allele-specific expression was analyzed by RT-PCR and Sanger sequencing. The direction of the cross is indicated on top of each panel, the embryonic stage on the left. Two replicates were analyzed for each stage and cross, which is represented by two individual sequencing chromatograms. The analyzed gene and the polymorphism between Col-0 and Ler are indicated in the grey box. Furthermore, the SNP is displayed in bold below each chromatogram. The sequenced reads from [33] for AT1G02780 in Ler x Col-0 2–4 cell and globular embryo libraries are indicated below the chromatograms on the right hand side. In addition, the allele-specific expression was assessed on F1 hybrid seedling cDNA libraries (8 days after sowing). (B) Allele-specific PCR was performed on genomic DNA extracted from hybrid F1 seedlings in order to test whether the assay amplifies both alleles with equal efficiency.
(PDF)

Figure S2 Allele-specific PCR on genomic DNA of hybrid F1 seedlings. A PCR covering the polymorphic region was performed on genomic DNA extracted from hybrid F1 seedlings and subsequently Sanger sequenced to test whether the assay amplifies both alleles with equal efficiency and is, thus, unbiased. We tested whether the assay introduces a technical bias towards one allele or the other for all MEG candidates (A) and PEG candidates (B) of this study. The analyzed gene and the polymorphism between Col-0 and Ler are indicated in the grey box beside the panels. Furthermore, the SNP is displayed in bold below each chromatogram.
(PDF)

Figure S3 Assessment of the quantitative nature of the Sanger sequencing approach. The allele specific expression assays of the 11 confirmed MEGs and the confirmed PEG were performed using a dilution series between Col-0 and Ler genomic DNA (9∶1, 3∶1, 1∶1, 1∶3, 1∶9). The analyzed gene and the polymorphism between Col-0 and Ler are indicated in the grey box beside the panels. Furthermore, the Col-0/Ler ratios are written at the top and the bottom of the figure.
(PDF)

Figure S4 Allele-specific expression analysis of partially confirmed MEGs and non-confirmed MEG candidates. Reciprocal hybrid embryos were isolated at 2.5 DAP (2–4 cell embryos) and at 4 DAP (globular embryos) and allele-specific expression was analyzed by RT-PCR and Sanger sequencing. The direction of the cross is indicated on top of each panel, the embryonic stage on the left. Two replicates were analyzed for each stage and cross, which is represented by two individual sequencing chromatograms. The analyzed gene and the polymorphism between Col-0 and Ler are indicated in the grey box atop of each panel. Furthermore, the polymorphic nucleotide is displayed in bold and underlined below each chromatogram. n.d. indicates that the transcript could not be amplified from the specific embryonic sample. (A) *AT3G21500*. (B) *AT2G01520*. (C) *AT1G20680*. (D) *AT5G51950*. (E) *AT1G29050*. (F) *AT3G44260* (shows biallelic expression in the 2–4 cell Col-0 x Ler replicate #1 and is, therefore, not confirmed as MEG) (G) *AT5G52060* (shows biallelic expression in the 2–4 cell Col-0 x Ler replicate #1 and is, therefore, not confirmed as MEG).
(PDF)

Figure S5 Allele-specific expression analysis of non-confirmed PEG candidates. Reciprocal hybrid embryos were isolated at 2.5 DAP (2–4 cell embryos) and at 4 DAP (globular embryos) and allele-specific expression was analyzed by RT-PCR and Sanger sequencing. The direction of the cross is indicated on top of each panel, the embryonic stage on the left. Two replicates were analyzed for each stage and cross, which is represented by two individual sequencing chromatograms. The analyzed gene and the polymorphism between Col-0 and Ler are indicated in the grey box atop of each panel. Furthermore, the polymorphic nucleotide is displayed in bold and underlined below each chromatogram. n.d. indicates that the transcript could not be amplified from the specific embryonic sample. (A) *AT2G20160* shows biallelic expression in 5 out of 8 samples. (B) *AT1G63260* shows biallelic expression in all samples from which the transcript was amplified.
(PDF)

Figure S6 Allele-specific expression analysis of extensively washed embryonic control samples. Allele-specific expression analysis of confirmed or partially confirmed MEGs (A) and confirmed (*AT3G26790*) and non-confirmed (*AT2G20160*) PEGs (B) in the 6× washed embryonic samples. The analyzed gene and the polymorphism between Col-0 and Ler are indicated in the grey box. The polymorphic nucleotide is displayed in bold and underlined below each chromatogram. The transcript of some genes could not be amplified from the 6× washed 2–4 cell Ler x Col-0 sample likely due to RNA degradation during extensive washing (indicated by n.d.). (C) Agarose gel analysis of the RT-PCR product of the partially confirmed MEG *AT1G29050* (left panel) and the non-confirmed *AT4G11960* (right panel).
(PDF)

Figure S7 MEG and PEG reporter line analysis on whole seeds. Whole siliques were stained for GUS expression over night and analyzed for GUS signals in the seed and embryo. Almost all MEG reporter lines show a more or less strong expression in the seed coat (A–G). Yet, the PEG reporter *FUS3::GUS* is specifically expressed in the embryo (H). Each panel depicts two strongly expressed T1 reporter lines that were used for further analysis. The reporter line is indicated on top of each panel and the individual line number in the upper right corner of each picture. Scale bar = 50 µm. (A) p*AT1G29660::GUS*. (B) *pAT1G72260::GUS*. (C) *pAT2G47115::GUS*. (D) *pAT5G62210::GUS*. (E) *pAT3G20520::GUS*. (F) *pAT2G17710::GUS*. (G) *pAT3G21500::GUS*. (H) *pAT3G26790::GUS* (*pFUS3::GUS*).
(PDF)

Figure S8 Embryo-specific expression of MEG reporter lines in isolated, self-fertilized embryos. Due to expression of all MEG reporters in the seed coat we isolated self-fertilized embryos carrying a MEG reporter line prior to GUS staining. Isolated embryos were released in GUS staining solution directly on a microscopic slide and were stained for 2–4 days at 37°C. 6 MEG reporter lines show a more or less strong and specific signal in the embryo. Each panel depicts two embryonic stages (early and late, indicated on the left), two independent lines (indicated on top) and a picture taken using DIC and bright-field microscopy (if not indicated otherwise). If no bright-field picture is shown (indicated by n.d.), then the signal was sufficiently visible when using DIC microscopy. Scale bar = 10 µm (A) p*AT1G29660::GUS*. (B) *pAT1G72260::GUS*. (C) *pAT2G47115::GUS*. (D) *pAT5G62210::GUS*. (E) *pAT3G20520::GUS*. (F) *pAT2G17710::GUS*.
(PDF)

Figure S9 Embryo-specific expression of *pAT3G21500::GUS*. *pAT3G21500::GUS* was the weakest line in terms of embryonic expression. The reporter line is expressed in self-fertilized 4 cell and 8 cell embryos but shows no or very weak expression only in earlier stages. This line was not included in the parent-of-origin-dependent reporter expression analysis. Scale bar = 10 µm.
(PDF)

Figure S10 Parent-of-origin-dependent expression analysis of the PEG reporter line *pFUS3::GUS* (*pAT3G26790::GUS*). (A) Quantifications of reciprocal crosses of two independent insertions of *pFUS3::GUS* (MR 398 and MR 399). First signals were detected 3 DAP, coinciding with the first signal in isolated (4-)8 cell stage embryos. At 3 DAP the reporter is expressed from both parents already. GUS signal of the maternally inherited reporter is depicted in black, whereas GUS signal of the paternally inherited reporter is in grey. Numbers of counted seeds are indicated above each column. (B) Reciprocally crossed and stained seeds are shown 2 DAP (upper row) and 4 DAP (lower row). Whereas no GUS signal can be detected at 2 DAP, the reporter is clearly expressed at 4 DAP. Scale bar = 50 µm. (C) Self-fertilized embryos were isolated at different time points and were stained on slide. Expression of the reporter was first detected at the (4-)8 cell stage. Scale bar = 10 µm.
(PDF)

Figure S11 Effect of PRC2 and *MET1* function on the imprinted expression in the embryo. Mutant embryonic samples were generated and the confirmed MEGs (A–E) and partially confirmed MEGs (F–J) were analyzed for derepression of the silent allele. Heterozygous *fie* mutants (in Col-0) were crossed maternally and paternally and heterozygous *met1-3* mutants (in Col-0) were crossed paternally to wild-type Ler as indicated above the chromatograms. Embryos were isolated at 2.5 DAP (2–4 cell embryos) and at 4 DAP (globular embryos, only for the cross *fie*/*FIE* x Ler). The embryonic stage is indicated on the left, the analyzed gene and the polymorphism between the mutant (all in Col-0 background) and the wild-type allele (Ler) is shown in the grey box beside each panel. Furthermore, the polymorphic nucleotide is displayed in bold and underlined below each chromatogram. NA indicates that the library was not available. (A) *AT2G47115*. (B) *AT5G62210*. (C) *AT3G20520*. (D) *AT2G17710*. (E) *AT3G21500*. (F) *AT2G01520*. (G) *AT1G20680*. (H) *AT5G51950*. (J) *AT1G29050*.
(PDF)

Table S1 All potentially imprinted candidate genes in the *Arabidopsis thaliana* embryo from [33]. Table S1a gives a list of all candidate MEGs and PEGs in the embryo called in this study. Table S1b gives an overview of which class of genes were called, tested and confirmed.
(XLS)

Table S2 Phenotype of T-DNA lines disrupting confirmed MEGs or the PEG. No obvious mutant phenotype was observed for any of the lines analyzed.
(PDF)

Table S3 Embryonic expression of confirmed and non-confirmed MEGs and PEGs (data from [45]).
(XLS)

Table S4 Parent-of-origin-dependent expression of confirmed MEGs and the PEG in other embryonic samples. One study analyzed torpedo-staged embryos in reciprocal crosses between Col-0 and Ler [28], whereas the second study analyzed early embryo stages (1–2 cell, 8 cell, 32 cell) from reciprocal crosses between Col-0 and Cvi [34].
(XLS)

Table S5 Parent-of-origin-dependent expression of confirmed embryonic MEGs and the embryonic PEG in the endosperm. Allele-specific and total reads of the embryonic MEGs and the PEG in the endosperm of reciprocal Col-0 x Ler hybrid seeds at 6 DAP [28].
(XLS)

Table S6 All primers used in this study.
(PDF)
