# Supplementary material for: Genomic Imprinting in the Arabidopsis Embryo Is Partly Regulated by PRC2
Source: PLoS Genet. 2013 Dec 5;9(12):e1003862. doi: 10.1371/journal.pgen.1003862 (PMC3854695; doi:10.1371/journal.pgen.1003862)
Supplement: Figure S1 — Allele-specific expression analysis of the biallelically expressed control gene AT1G02780. (A) Reciprocal hybrid embryos were isolated at 2.5 DAP (2–4 cell embryos) and at 4 DAP (globular embryos) and allele-specific expression was analyzed by RT-PCR and Sanger sequencing. The direction of the cross is indicated on top of each panel, the embryonic stage on the left. Two replicates were analyzed for each stage and cross, which is represented by two individual sequencing chromatograms. The analyzed gene and the polymorphism between Col-0 and Ler are indicated in the grey box. Furthermore, the SNP is displayed in bold below each chromatogram. The sequenced reads from [33] for AT1G02780 in Ler x Col-0 2–4 cell and globular embryo libraries are indicated below the chromatograms on the right hand side. In addition, the allele-specific expression was assessed on F1 hybrid seedling cDNA libraries (8 days after sowing). (B) Allele-specific PCR was performed on genomic DNA extracted from hybrid F1 seedlings in order to test whether the assay amplifies both alleles with equal efficiency. (PDF) [file pgen.1003862.s001.pdf]

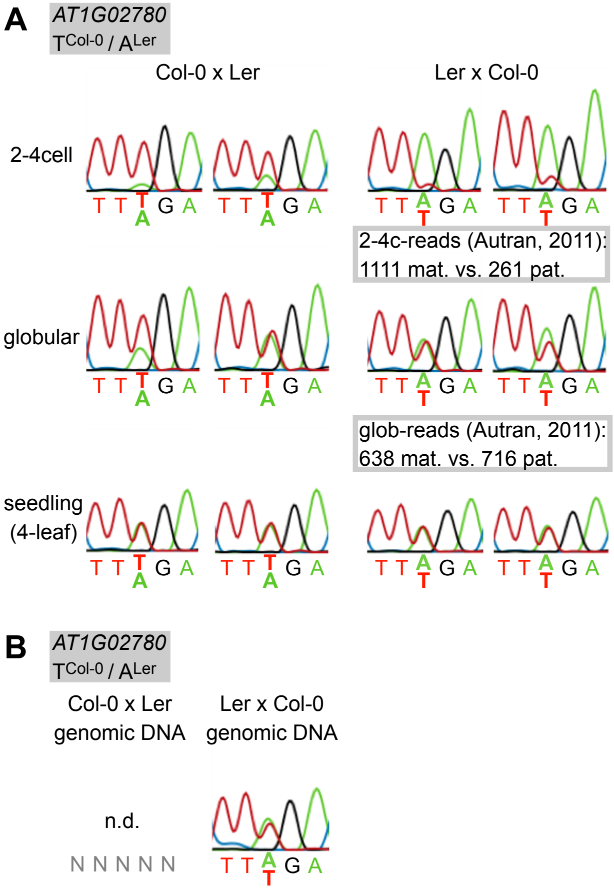
A
AT1G02780
TCol-0 / ALer
Col-0 x Ler
Ler x Col-0
2-4cell
T T T/A G A
T T A/T G A
2-4c-reads (Autran, 2011):
1111 mat. vs. 261 pat.
globular
glob-reads (Autran, 2011):
638 mat. vs. 716 pat.
seedling
(4-leaf)
B
AT1G02780
TCol-0 / ALer
Col-0 x Ler
genomic DNA
Ler x Col-0
genomic DNA
n.d.
N N N N N
