# Supplementary figures and images for: Genomic Imprinting in the Arabidopsis Embryo Is Partly Regulated by PRC2
Source: PLoS Genet. 2013 Dec 5;9(12):e1003862. doi: 10.1371/journal.pgen.1003862 (PMC3854695; doi:10.1371/journal.pgen.1003862)

**A** Col-0 x Ler genomic DNA Ler x Col-0 genomic DNA

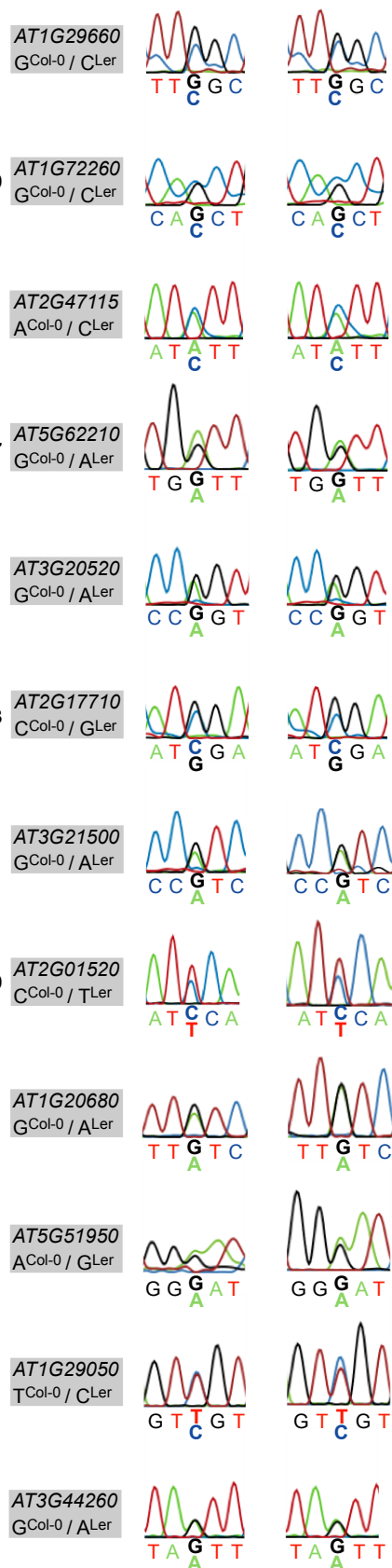

**B** Col-0 x Ler genomic DNA Ler x Col-0 genomic DNA

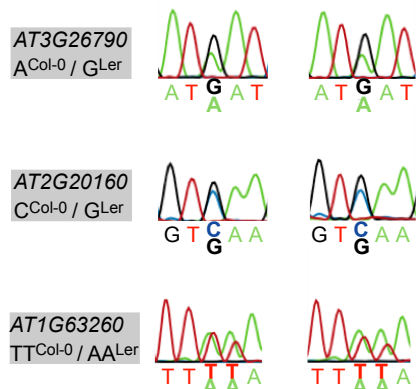

Supplement: Figure S2 — Allele-specific PCR on genomic DNA of hybrid F1 seedlings. A PCR covering the polymorphic region was performed on genomic DNA extracted from hybrid F1 seedlings and subsequently Sanger sequenced to test whether the assay amplifies both alleles with equal efficiency and is, thus, unbiased. We tested whether the assay introduces a technical bias towards one allele or the other for all MEG candidates (A) and PEG candidates (B) of this study. The analyzed gene and the polymorphism between Col-0 and Ler are indicated in the grey box beside the panels. Furthermore, the SNP is displayed in bold below each chromatogram. (PDF) [file pgen.1003862.s002.pdf]

**A***AT2G20160**C*<sup>Col-0</sup> / *G*<sup>Ler</sup>

Col-0 x Ler

Ler x Col-0

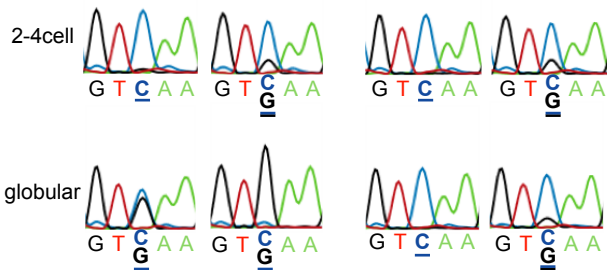**B***AT1G63260**TT*<sup>Col-0</sup> / *AA*<sup>Ler</sup>

Col-0 x Ler

Ler x Col-0

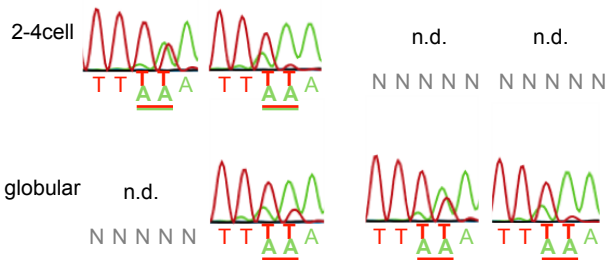

Supplement: Figure S5 — Allele-specific expression analysis of non-confirmed PEG candidates. Reciprocal hybrid embryos were isolated at 2.5 DAP (2–4 cell embryos) and at 4 DAP (globular embryos) and allele-specific expression was analyzed by RT-PCR and Sanger sequencing. The direction of the cross is indicated on top of each panel, the embryonic stage on the left. Two replicates were analyzed for each stage and cross, which is represented by two individual sequencing chromatograms. The analyzed gene and the polymorphism between Col-0 and Ler are indicated in the grey box atop of each panel. Furthermore, the polymorphic nucleotide is displayed in bold and underlined below each chromatogram. n.d. indicates that the transcript could not be amplified from the specific embryonic sample. (A) AT2G20160 shows biallelic expression in 5 out of 8 samples. (B) AT1G63260 shows biallelic expression in all samples from which the transcript was amplified. (PDF) [file pgen.1003862.s005.pdf]

**A**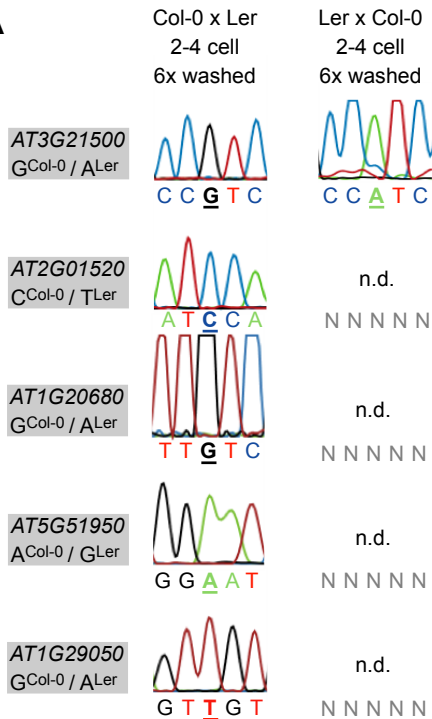**B**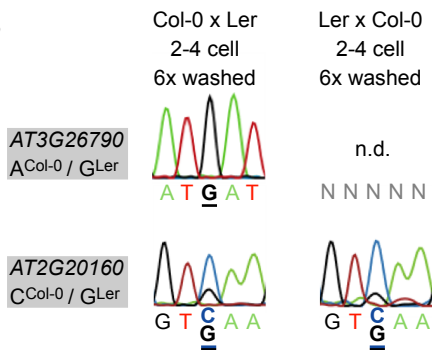**C**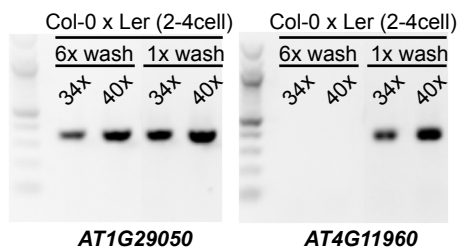

Supplement: Figure S6 — Allele-specific expression analysis of extensively washed embryonic control samples. Allele-specific expression analysis of confirmed or partially confirmed MEGs (A) and confirmed (AT3G26790) and non-confirmed (AT2G20160) PEGs (B) in the 6× washed embryonic samples. The analyzed gene and the polymorphism between Col-0 and Ler are indicated in the grey box. The polymorphic nucleotide is displayed in bold and underlined below each chromatogram. The transcript of some genes could not be amplified from the 6× washed 2–4 cell Ler x Col-0 sample likely due to RNA degradation during extensive washing (indicated by n.d.). (C) Agarose gel analysis of the RT-PCR product of the partially confirmed MEG AT1G29050 (left panel) and the non-confirmed AT4G11960 (right panel). (PDF) [file pgen.1003862.s006.pdf]

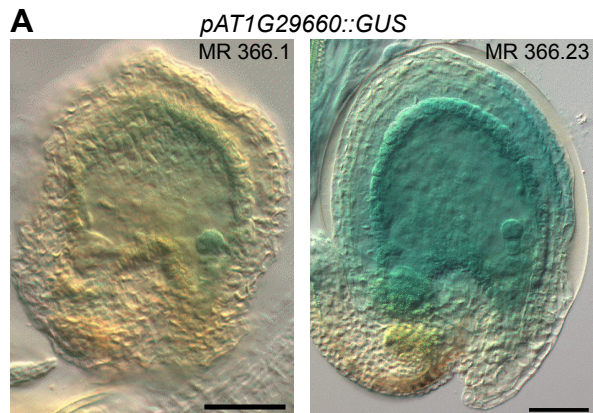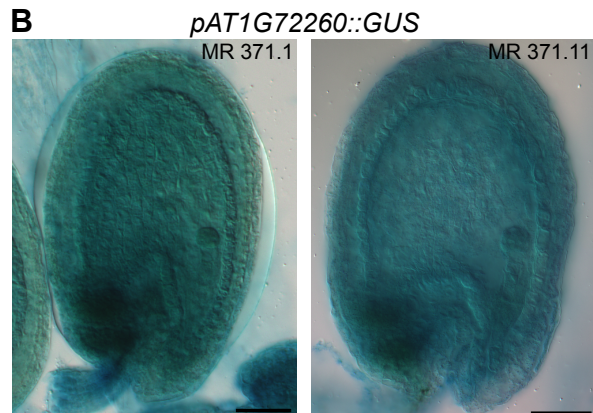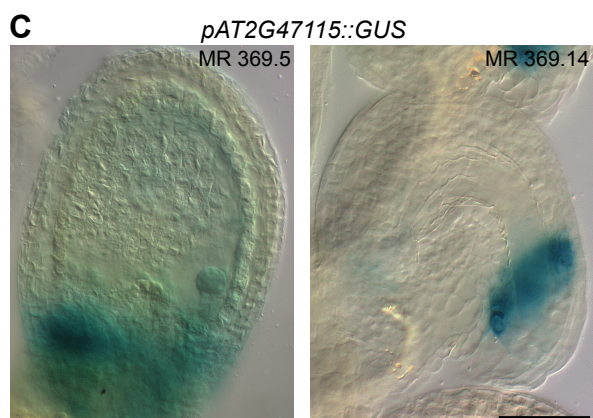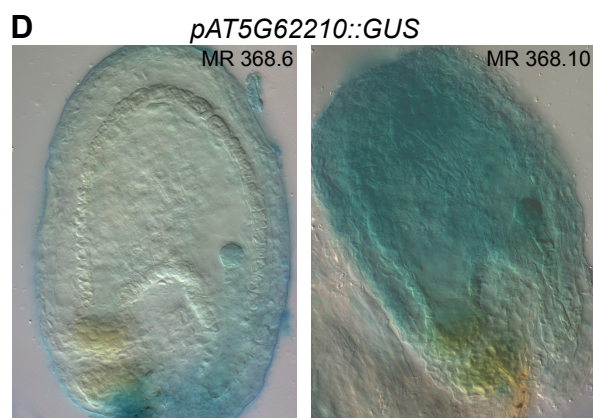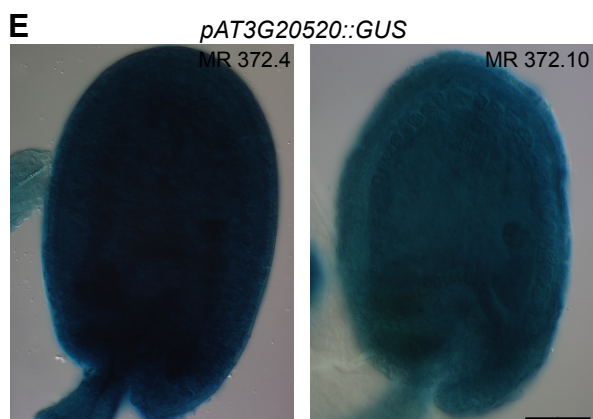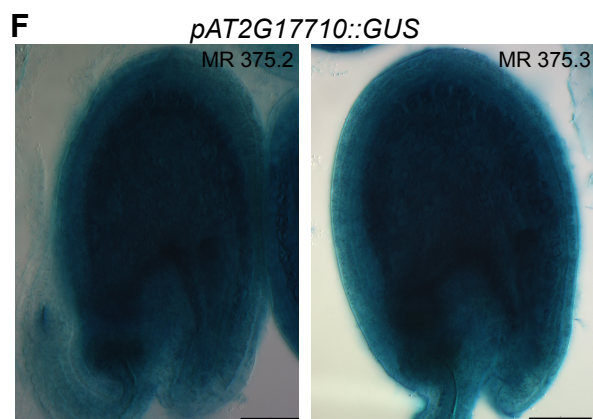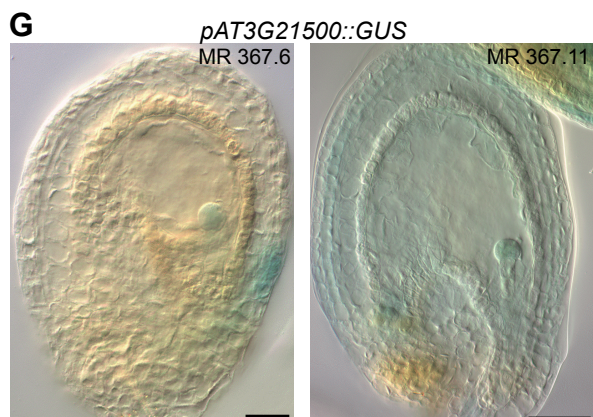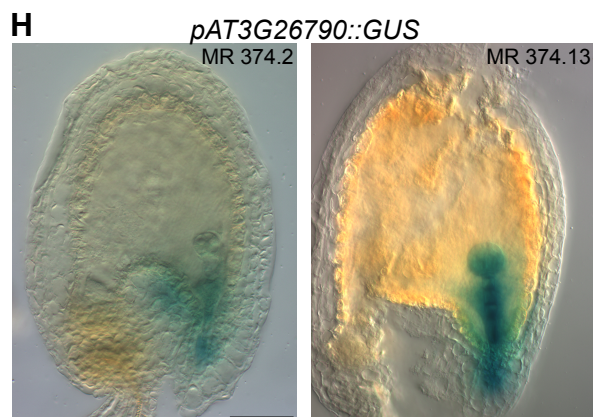

Supplement: Figure S7 — MEG and PEG reporter line analysis on whole seeds. Whole siliques were stained for GUS expression over night and analyzed for GUS signals in the seed and embryo. Almost all MEG reporter lines show a more or less strong expression in the seed coat (A–G). Yet, the PEG reporter FUS3::GUS is specifically expressed in the embryo (H). Each panel depicts two strongly expressed T1 reporter lines that were used for further analysis. The reporter line is indicated on top of each panel and the individual line number in the upper right corner of each picture. Scale bar = 50 µm. (A) pAT1G29660::GUS. (B) pAT1G72260::GUS. (C) pAT2G47115::GUS. (D) pAT5G62210::GUS. (E) pAT3G20520::GUS. (F) pAT2G17710::GUS. (G) pAT3G21500::GUS. (H) pAT3G26790::GUS (pFUS3::GUS). (PDF) [file pgen.1003862.s007.pdf]

*pAT3G21500::GUS*

2cell

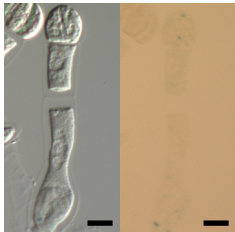

4cell

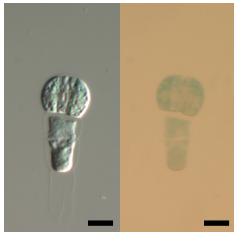

8cell

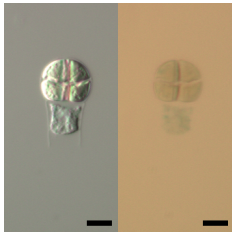

Supplement: Figure S9 — Embryo-specific expression of pAT3G21500::GUS. pAT3G21500::GUS was the weakest line in terms of embryonic expression. The reporter line is expressed in self-fertilized 4 cell and 8 cell embryos but shows no or very weak expression only in earlier stages. This line was not included in the parent-of-origin-dependent reporter expression analysis. Scale bar = 10 µm. (PDF) [file pgen.1003862.s009.pdf]

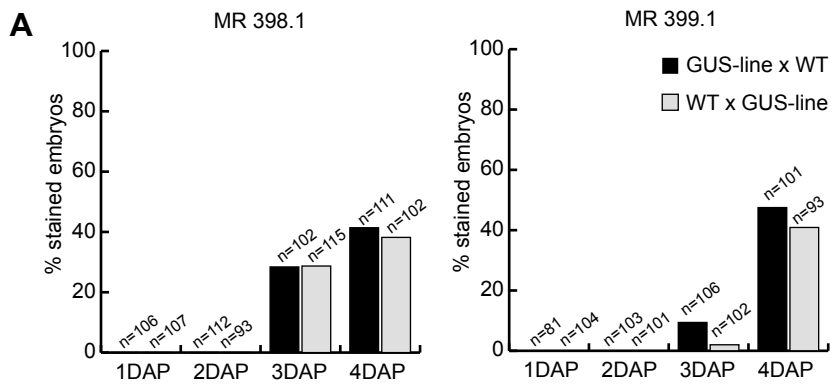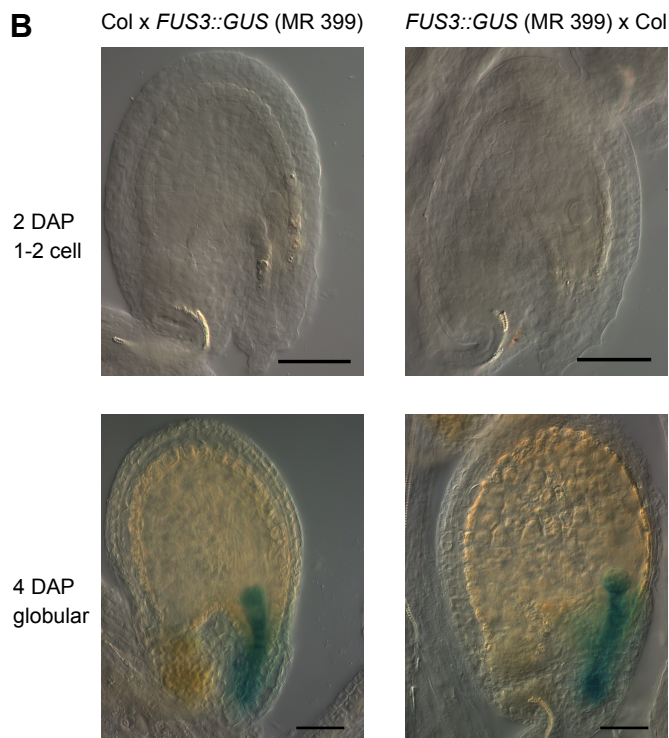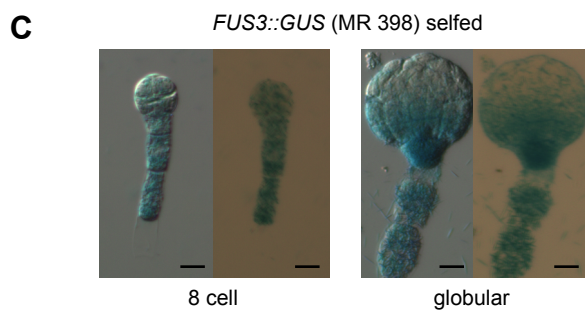

Supplement: Figure S10 — Parent-of-origin-dependent expression analysis of the PEG reporter line pFUS3::GUS (pAT3G26790::GUS). (A) Quantifications of reciprocal crosses of two independent insertions of pFUS3::GUS (MR 398 and MR 399). First signals were detected 3 DAP, coinciding with the first signal in isolated (4-)8 cell stage embryos. At 3 DAP the reporter is expressed from both parents already. GUS signal of the maternally inherited reporter is depicted in black, whereas GUS signal of the paternally inherited reporter is in grey. Numbers of counted seeds are indicated above each column. (B) Reciprocally crossed and stained seeds are shown 2 DAP (upper row) and 4 DAP (lower row). Whereas no GUS signal can be detected at 2 DAP, the reporter is clearly expressed at 4 DAP. Scale bar = 50 µm. (C) Self-fertilized embryos were isolated at different time points and were stained on slide. Expression of the reporter was first detected at the (4-)8 cell stage. Scale bar = 10 µm. (PDF) [file pgen.1003862.s010.pdf]

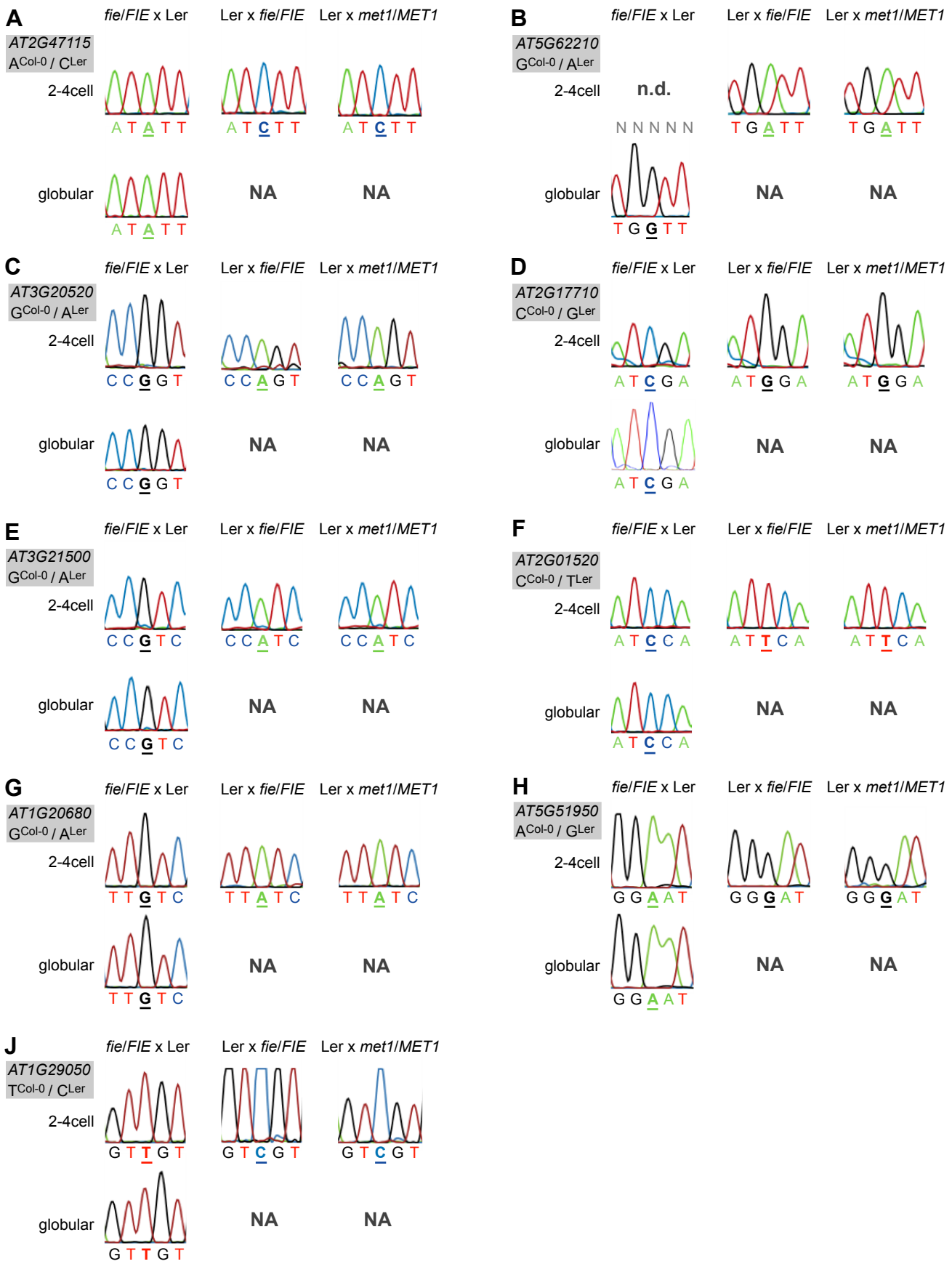

Supplement: Figure S11 — Effect of PRC2 and MET1 function on the imprinted expression in the embryo. Mutant embryonic samples were generated and the confirmed MEGs (A–E) and partially confirmed MEGs (F–J) were analyzed for derepression of the silent allele. Heterozygous fie mutants (in Col-0) were crossed maternally and paternally and heterozygous met1-3 mutants (in Col-0) were crossed paternally to wild-type Ler as indicated above the chromatograms. Embryos were isolated at 2.5 DAP (2–4 cell embryos) and at 4 DAP (globular embryos, only for the cross fie/FIE x Ler). The embryonic stage is indicated on the left, the analyzed gene and the polymorphism between the mutant (all in Col-0 background) and the wild-type allele (Ler) is shown in the grey box beside each panel. Furthermore, the polymorphic nucleotide is displayed in bold and underlined below each chromatogram. NA indicates that the library was not available. (A) AT2G47115. (B) AT5G62210. (C) AT3G20520. (D) AT2G17710. (E) AT3G21500. (F) AT2G01520. (G) AT1G20680. (H) AT5G51950. (J) AT1G29050. (PDF) [file pgen.1003862.s011.pdf]
