# Supplementary material for: Genomic Imprinting in the Arabidopsis Embryo Is Partly Regulated by PRC2
Source: PLoS Genet. 2013 Dec 5;9(12):e1003862. doi: 10.1371/journal.pgen.1003862 (PMC3854695; doi:10.1371/journal.pgen.1003862)
Supplement: Figure S3 — Assessment of the quantitative nature of the Sanger sequencing approach. The allele specific expression assays of the 11 confirmed MEGs and the confirmed PEG were performed using a dilution series between Col-0 and Ler genomic DNA (9∶1, 3∶1, 1∶1, 1∶3, 1∶9). The analyzed gene and the polymorphism between Col-0 and Ler are indicated in the grey box beside the panels. Furthermore, the Col-0/Ler ratios are written at the top and the bottom of the figure. (PDF) [file pgen.1003862.s003.pdf]

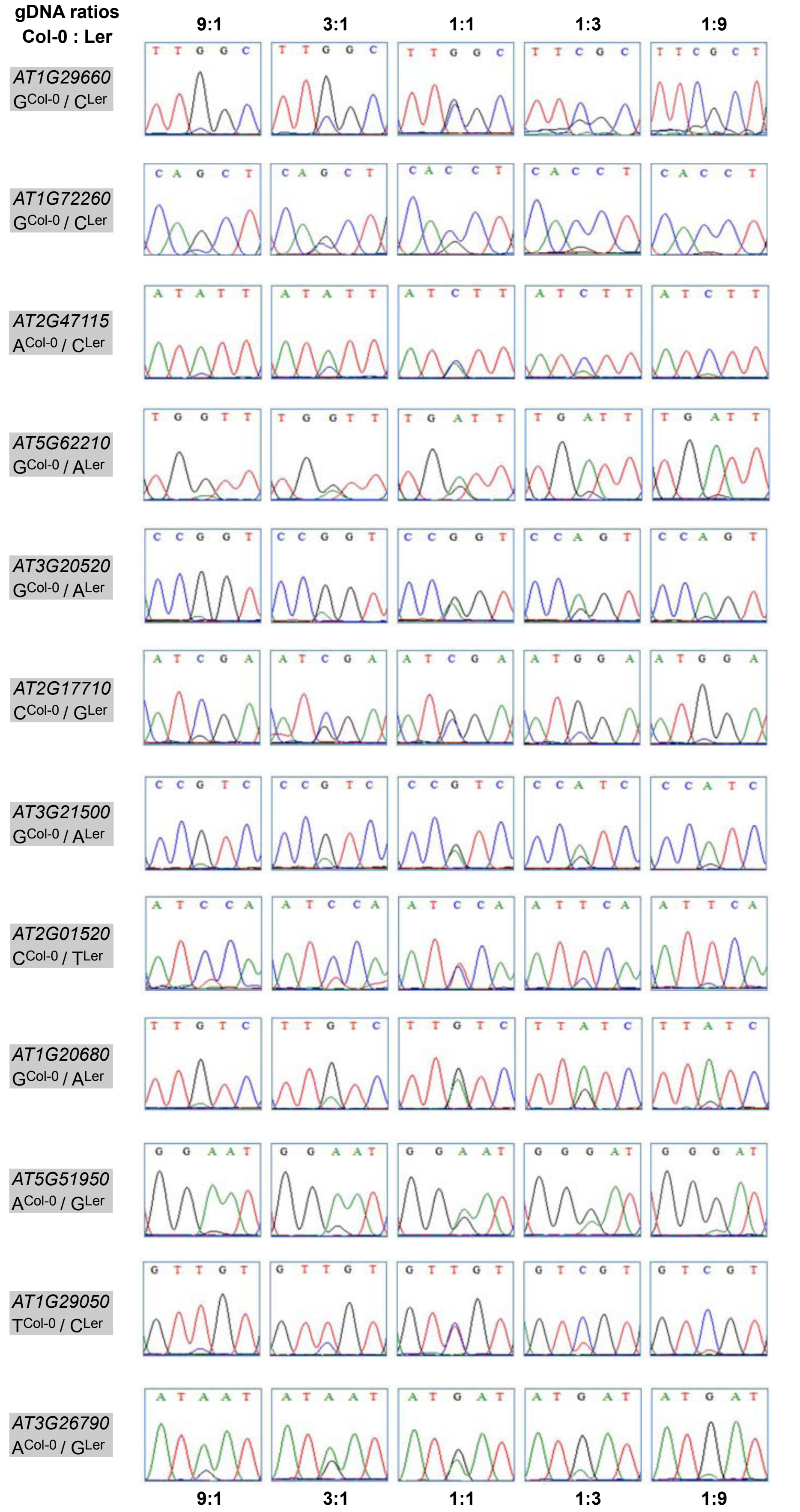

gDNA ratios
Col-0 : Ler
9:1
3:1
1:1
1:3
1:9
AT1G29660
G^{Col-0} / C^{Ler}
AT1G72260
G^{Col-0} / C^{Ler}
AT2G47115
A^{Col-0} / C^{Ler}
AT5G62210
G^{Col-0} / A^{Ler}
AT3G20520
G^{Col-0} / A^{Ler}
AT2G17710
C^{Col-0} / G^{Ler}
AT3G21500
G^{Col-0} / A^{Ler}
AT2G01520
C^{Col-0} / T^{Ler}
AT1G20680
G^{Col-0} / A^{Ler}
AT5G51950
A^{Col-0} / G^{Ler}
AT1G29050
T^{Col-0} / C^{Ler}
AT3G26790
A^{Col-0} / G^{Ler}
9:1
3:1
1:1
1:3
1:9
