# Supplementary material for: Genomic Imprinting in the Arabidopsis Embryo Is Partly Regulated by PRC2
Source: PLoS Genet. 2013 Dec 5;9(12):e1003862. doi: 10.1371/journal.pgen.1003862 (PMC3854695; doi:10.1371/journal.pgen.1003862)
Supplement: Figure S4 — Allele-specific expression analysis of partially confirmed MEGs and non-confirmed MEG candidates. Reciprocal hybrid embryos were isolated at 2.5 DAP (2–4 cell embryos) and at 4 DAP (globular embryos) and allele-specific expression was analyzed by RT-PCR and Sanger sequencing. The direction of the cross is indicated on top of each panel, the embryonic stage on the left. Two replicates were analyzed for each stage and cross, which is represented by two individual sequencing chromatograms. The analyzed gene and the polymorphism between Col-0 and Ler are indicated in the grey box atop of each panel. Furthermore, the polymorphic nucleotide is displayed in bold and underlined below each chromatogram. n.d. indicates that the transcript could not be amplified from the specific embryonic sample. (A) AT3G21500. (B) AT2G01520. (C) AT1G20680. (D) AT5G51950. (E) AT1G29050. (F) AT3G44260 (shows biallelic expression in the 2–4 cell Col-0 x Ler replicate #1 and is, therefore, not confirmed as MEG) (G) AT5G52060 (shows biallelic expression in the 2–4 cell Col-0 x Ler replicate #1 and is, therefore, not confirmed as MEG). (PDF) [file pgen.1003862.s004.pdf]

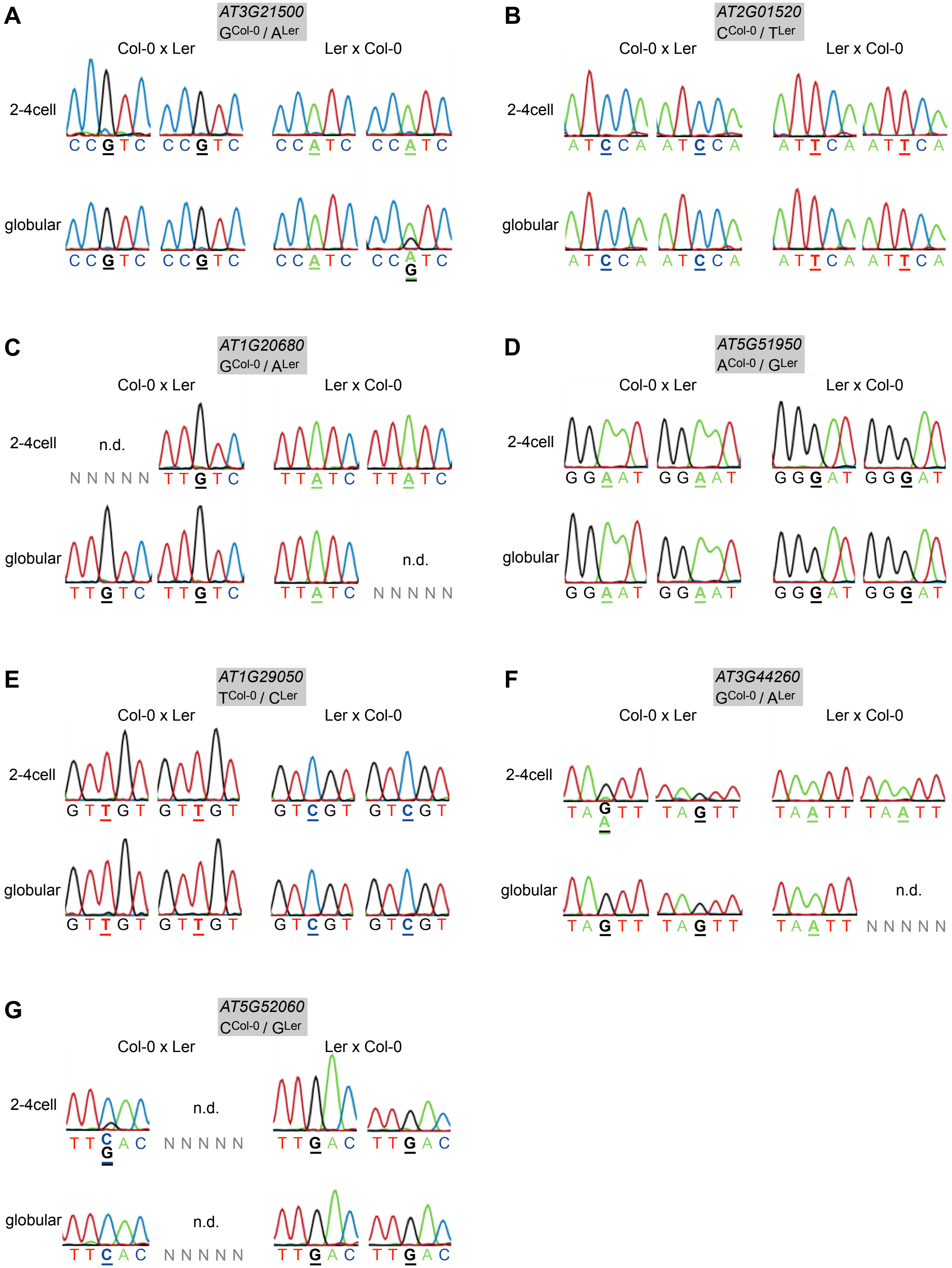

A
AT3G21500
GCol-0 / ALer
Col-0 x Ler
Ler x Col-0
2-4cell
globular
C C G T C
C C A T C
C C A/G T C
B
AT2G01520
CCol-0 / TLer
Col-0 x Ler
Ler x Col-0
2-4cell
globular
A T C C A
A T T C A
C
AT1G20680
GCol-0 / ALer
Col-0 x Ler
Ler x Col-0
2-4cell
globular
n.d.
N N N N N
T T G T C
T T A T C
D
AT5G51950
ACol-0 / GLer
Col-0 x Ler
Ler x Col-0
2-4cell
globular
G G A A T
G G G A T
E
AT1G29050
TCol-0 / CLer
Col-0 x Ler
Ler x Col-0
2-4cell
globular
G T T G T
G T C G T
F
AT3G44260
GCol-0 / ALer
Col-0 x Ler
Ler x Col-0
2-4cell
globular
T A G/A T T
T A G T T
T A A T T
n.d.
N N N N N
G
AT5G52060
CCol-0 / GLer
Col-0 x Ler
Ler x Col-0
2-4cell
globular
T T C/G A C
T T C A C
n.d.
N N N N N
T T G A C
