# Supplementary material for: Genomic Imprinting in the Arabidopsis Embryo Is Partly Regulated by PRC2
Source: PLoS Genet. 2013 Dec 5;9(12):e1003862. doi: 10.1371/journal.pgen.1003862 (PMC3854695; doi:10.1371/journal.pgen.1003862)
Supplement: Figure S8 — Embryo-specific expression of MEG reporter lines in isolated, self-fertilized embryos. Due to expression of all MEG reporters in the seed coat we isolated self-fertilized embryos carrying a MEG reporter line prior to GUS staining. Isolated embryos were released in GUS staining solution directly on a microscopic slide and were stained for 2–4 days at 37°C. 6 MEG reporter lines show a more or less strong and specific signal in the embryo. Each panel depicts two embryonic stages (early and late, indicated on the left), two independent lines (indicated on top) and a picture taken using DIC and bright-field microscopy (if not indicated otherwise). If no bright-field picture is shown (indicated by n.d.), then the signal was sufficiently visible when using DIC microscopy. Scale bar = 10 µm (A) pAT1G29660::GUS. (B) pAT1G72260::GUS. (C) pAT2G47115::GUS. (D) pAT5G62210::GUS. (E) pAT3G20520::GUS. (F) pAT2G17710::GUS. (PDF) [file pgen.1003862.s008.pdf]

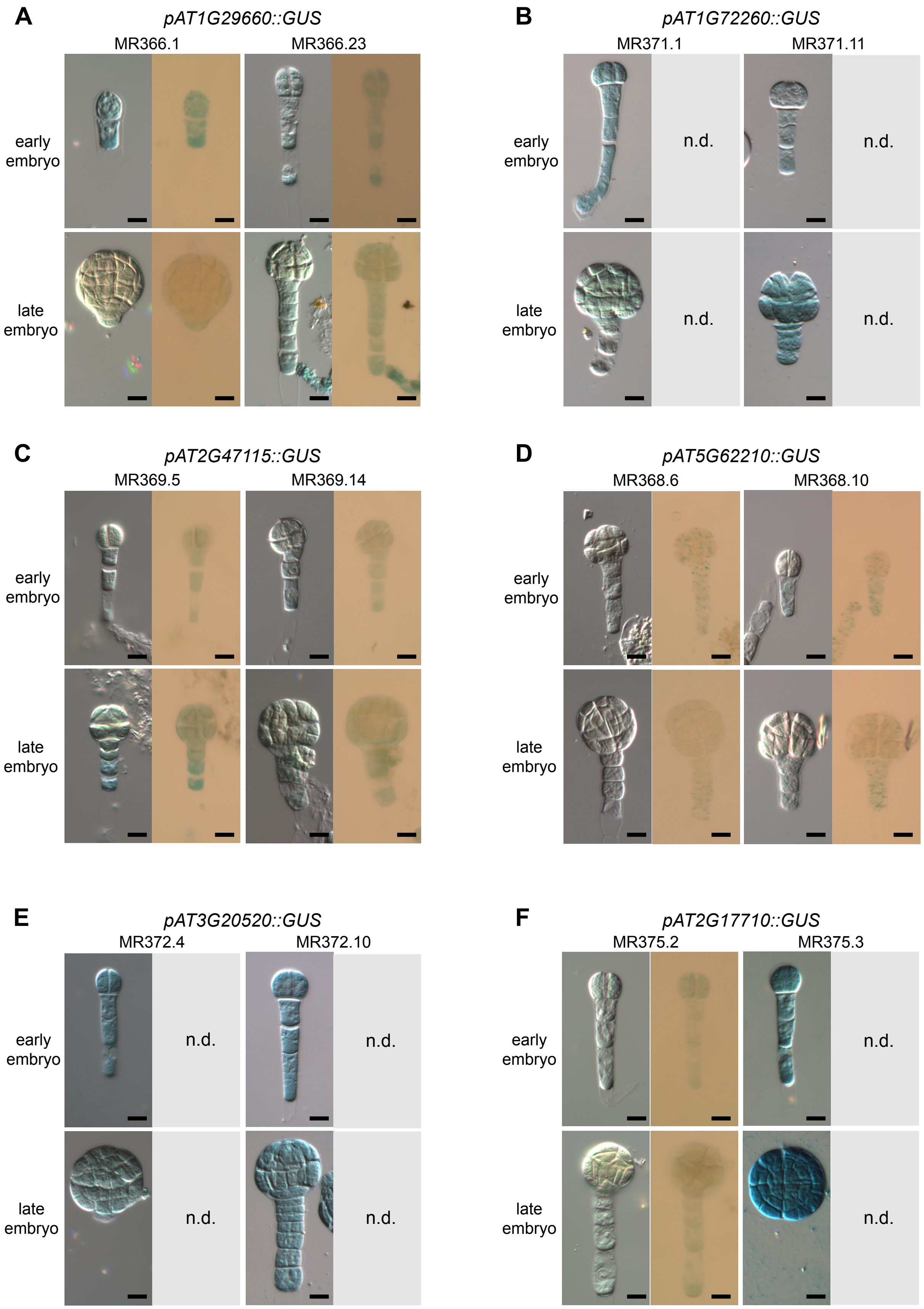

A
pAT1G29660::GUS
MR366.1
MR366.23
early embryo
late embryo
B
pAT1G72260::GUS
MR371.1
MR371.11
n.d.
C
pAT2G47115::GUS
MR369.5
MR369.14
D
pAT5G62210::GUS
MR368.6
MR368.10
E
pAT3G20520::GUS
MR372.4
MR372.10
F
pAT2G17710::GUS
MR375.2
MR375.3
