# Supplementary material for: Genomic Imprinting in the Arabidopsis Embryo Is Partly Regulated by PRC2
Source: PLoS Genet. 2013 Dec 5;9(12):e1003862. doi: 10.1371/journal.pgen.1003862 (PMC3854695; doi:10.1371/journal.pgen.1003862)
Supplement: Table S2 — Phenotype of T-DNA lines disrupting confirmed MEGs or the PEG. No obvious mutant phenotype was observed for any of the lines analyzed. (PDF) [file pgen.1003862.s013.pdf]

Table S2. Phenotyping of T-DNA lines disrupting confirmed MEGs or the PEG. No obvious mutant phenotype was observed.

T-DNA line	locus	insertion site	seed set[1]	embryo patterning[2]	comments
SAIL_641_B05	*AT1G29660*	1st exon	wild-type	wild-type	MEG
SALK_023064C	*AT1G29660*	5th exon	wild-type	wild-type	MEG
SALK_039929C	*AT1G72260*	promoter	wild-type	wild-type	MEG
GABI_054H02	*AT1G72260*	3rd exon	wild-type	wild-type	MEG
FLAG_382E11	*AT2G47115*	5th exon	wild-type	wild-type	MEG
SALK_038225	*AT5G62210*	2nd exon	wild-type	wild-type	MEG
GABI_243D08	*AT5G62210*	intron	wild-type	wild-type	MEG
SAIL_330_H06	*AT5G62210*	promoter	wild-type	wild-type	MEG
SALK_010358.23.30	*AT3G20520*	2nd exon	wild-type	wild-type	MEG
SALK_152374.35.55	*AT3G20520*	4th exon	wild-type	wild-type	MEG
SALK 067511	*AT2G17710*	promoter	wild-type	wild-type	MEG
SALK 067639	*AT2G17710*	promoter	wild-type	wild-type	MEG
SALK_112762	*AT3G21500*	4th exon	wild-type	wild-type	MEG
SALK_039162.21.10	*AT3G26790*	1st exon	wild-type	wild-type	PEG; late phenotype (Raz et al. 2001)[3]

[1] mature siliques of 12-24 genotyped individuals were opened and examined for aborted seeds or infertile ovules.
[2] siliques at different stages were cleared using Hoyer's solution and analyzed for patterning defects.
[3] Raz et al. 2001 find a prolonged cell division phase in late embryos (torpedo stage). We analyzed earlier stages only.
