# Supplementary material for: Genomic Imprinting in the Arabidopsis Embryo Is Partly Regulated by PRC2
Source: PLoS Genet. 2013 Dec 5;9(12):e1003862. doi: 10.1371/journal.pgen.1003862 (PMC3854695; doi:10.1371/journal.pgen.1003862)
Supplement: Table S6 — All primers used in this study. (PDF) [file pgen.1003862.s017.pdf]

Table S6. All primers used in this study.

priMR number	sequence (5'-3')	general use	description
priMR 43	TCCAAATCCTCCAATGGAAC	RT-PCR & Sanger	AT3G21500
priMR 44	TGACCGTCCTTCTTGCTTTC	RT-PCR & Sanger	AT3G21500
priMR 45	AAGATCGTCACCGAGGTCTG	RT-PCR & Sanger	AT3G44260
priMR 46	TCGTGACTCGTGAAGTCTGG	RT-PCR & Sanger	AT3G44260
priMR 49	TTCACTCGCTACACAAGGAAGA	RT-PCR & Sanger	AT2G01520
priMR 50	TCGTTTTGCTTCTCCCAGAT	RT-PCR & Sanger	AT2G01520
priMR 51	TGGGAAACAGGAGTTCTTGG	RT-PCR & Sanger	AT2G47115
priMR 52	TGCTGACGGACATTGTGTTT	RT-PCR & Sanger	AT2G47115
priMR 53	GCAAAGCTAACAACGGAACC	RT-PCR & Sanger	AT1G20680
priMR 54	TGGCAGAATCTGTAGAATCGAG	RT-PCR & Sanger	AT1G20680
priMR 57	GCTCTCGTGGTAGCATTCAA	RT-PCR & Sanger	AT5G51950
priMR 58	CGATGGTAGGGCAAGGTATG	RT-PCR & Sanger	AT5G51950
priMR 59	TTCATGCCTCAGTTCTACTCCA	RT-PCR & Sanger	AT1G29660
priMR 60	GCGCCATAAGCATTGATGTA	RT-PCR & Sanger	AT1G29660
priMR 61	CCCAGGTTATTTCTGGGAAG	RT-PCR & Sanger	At1G24090 / At1G24095
priMR 62	GGCTCAGCTGCTTTAGACTG	RT-PCR & Sanger	At1G24090 / At1G24095
priMR 63	GCTGCGTAGAGAAGTTGGTTC	RT-PCR & Sanger	AT1G29050
priMR 64	GGGTTGATCAAAACGTCGAT	RT-PCR & Sanger	AT1G29050
priMR 65	AAAGAATATGCTCTTGTGTATTTGAA	RT-PCR & Sanger	AT5G42650
priMR 66	ATAAACAATGCGCCGGTAAG	RT-PCR & Sanger	AT5G42650
priMR 115	GTGGTTCAAACCGGAGACAT	RT-PCR & Sanger	AT5G42650
priMR 116	GGGTGGATGAAGATTTTGTT	RT-PCR & Sanger	AT5G42650
priMR 67	CAAGATGGTAAGATTGGGATAGTG	RT-PCR & Sanger	AT2G44470
priMR 68	GGAGATGAGCGGTGAAGTGT	RT-PCR & Sanger	AT2G44470
priMR 71	TCATCCTTGAAAAGGAGACAAA	RT-PCR & Sanger	AT3G20520
priMR 72	GAGAAGAAGCAGGAGGCTGA	RT-PCR & Sanger	AT3G20520
priMR 117	AAGTGTTACGGCGAATTTGG	RT-PCR & Sanger	AT3G20520
priMR 118	TTTTCCTCTGCCTTTGATGG	RT-PCR & Sanger	AT3G20520
priMR 73	TCTCTTTAGTTTGAAGGTTAGATTCAG	RT-PCR & Sanger	AT2G17710
priMR 74	TGATCTTCGGTTTCCAGGTC	RT-PCR & Sanger	AT2G17710
priMR 137	CGCTTCTTCCTCCATCTCAG	RT-PCR & Sanger	AT2G17710
priMR 138	ACACGAGCTGCAGCCTTAAT	RT-PCR & Sanger	AT2G17710
priMR 77	AACATGTACCTGCGAGCAAA	RT-PCR & Sanger	AT3G58780
priMR 78	TTTGGATCTCGACGTGTTTT	RT-PCR & Sanger	AT3G58780
priMR 79	GAGTGTTCATGGCACCACAC	RT-PCR & Sanger	AT1G72260
priMR 80	TCCCTTGTGATCAAACAAGTAAA	RT-PCR & Sanger	AT1G72260
priMR 81	TGTTGTGGTTTGGCAATCTT	RT-PCR & Sanger	AT1G63260
priMR 82	GTTATCCTGCGGTGAATGCT	RT-PCR & Sanger	AT1G63260
priMR 103	CTGTTGTGGTTTGGCAATCTT	RT-PCR & Sanger	AT1G63260
priMR 104	GCGATCTTCAATGTGGTCCT	RT-PCR & Sanger	AT1G63260
priMR 168	GGAGAATCCGGTGAAGTGTT	RT-PCR & Sanger	AT1G63260
priMR 169	GCCAAACCACAACAGTACCA	RT-PCR & Sanger	AT1G63260
priMR 83	TGCGAAGCTCTTCTTCTTCC	RT-PCR & Sanger	AT2G20160
priMR 84	CAGATGGTAGCGCACATGAT	RT-PCR & Sanger	AT2G20160
priMR 135	TGCGAAGCTCTTCTTCTTCC	RT-PCR & Sanger	AT2G20160
priMR 136	CGCAGAGTTCGTGAAAAACA	RT-PCR & Sanger	AT2G20160
priMR 85	CACTGGCTCATCTCCACATTT	RT-PCR & Sanger	AT2G24430
priMR 86	CGACTTCACTCCAAGTCCTCA	RT-PCR & Sanger	AT2G24430
priMR 105	AAGTCCATGCACTGGCTCAT	RT-PCR & Sanger	AT2G24430
priMR 106	AGCACCACCATGGACTCTTC	RT-PCR & Sanger	AT2G24430
priMR 87	AAGTGCTTGGTGAGCAACAG	RT-PCR & Sanger	AT3G63480
priMR 88	AGTAGAAGCCACTGCGCCTA	RT-PCR & Sanger	AT3G63480
priMR 107	GCATCCTGTAAGATGCGAGT	RT-PCR & Sanger	AT3G63480
priMR 108	TGGCTCAGAGAAAGCAGACA	RT-PCR & Sanger	AT3G63480
priMR 89	CATACGTCGGAGAGAGCTGA	RT-PCR & Sanger	AT3G26790
priMR 90	AAACCCAAAGAGATCCACCA	RT-PCR & Sanger	AT3G26790
priMR 109	CACCATCAAGAATACCATGAGC	RT-PCR & Sanger	AT3G26790
priMR 110	GACACGACGGCTCTTACCTC	RT-PCR & Sanger	AT3G26790
priMR 93	CTTTCCTAATGAGAGCAAAGTTTC	RT-PCR & Sanger	AT4G11960
priMR 94	TCCCCAATGATAGTAACACCAA	RT-PCR & Sanger	AT4G11960
priMR 95	CATGCTTCCTTTCTCGGCTA	RT-PCR & Sanger	AT1G02780 (BiG)
priMR 96	ATGAAGGGTCGTCACTCTGG	RT-PCR & Sanger	AT1G02780 (BiG)
priMR 97	ACGCAACAACCAAACTCTGA	RT-PCR & Sanger	AT5G62210
priMR 98	GAAGGTTCCAAATCCGTGAA	RT-PCR & Sanger	AT5G62210
priMR 99	CTCCAACCAAAAGACCTTGG	RT-PCR & Sanger	AT1G57800
priMR 100	CCCCGTATCAACTTGTCCAT	RT-PCR & Sanger	AT1G57800
priMR 113	TCTTGCCACTGGAAGCATTT	RT-PCR & Sanger	AT1G57800
priMR 114	CCCCGTATCAACTTGTCCAT	RT-PCR & Sanger	AT1G57800
priMR 101	GGCTCCTCTTCAATCTCTTGC	RT-PCR & Sanger	AT5G52060
priMR 102	TGTCAAGTTACAAAGAAAGATGCAG	RT-PCR & Sanger	AT5G52060
priMR 119	TCAAAGTTCTGCAGATTATCCG	genotyping	SALK_112762.41.75 = 43-1
priMR 120	TTCCACAATTAGATTGCAGCC	genotyping	SALK_112762.41.75 = 43-1
priMR 121	TAGATCGAACAACCGTGGTTC	genotyping	FLAG_382E11 = 51-1
priMR 122	GGGATATGAACAATTGCCATG	genotyping	FLAG_382E11 = 51-1
priMR 123	GAAAACACAGCTGCCTAGCTG	genotyping	SAIL_641_B05 = 59-1
priMR 124	CTTCTCTGATTCCAGCAGCTG	genotyping	SAIL_641_B05 = 59-1
priMR 125	ATTCGATGGAAAACTATGGGG	genotyping	SALK_023064.22.75 = 59-2
priMR 126	TAGATCCAGTTTCAGCGAAGC	genotyping	SALK_023064.22.75 = 59-2
priMR 127	ACGCTCCATTCACAATTTCAC	genotyping	SALK_039929.55.25 = 79-1
priMR 128	GAACTCTATGCGTGCCAAGAG	genotyping	SALK_039929.55.25 = 79-1
priMR 129	TGTATTTTCATGGTGTGTGGTTG	genotyping	SALK_038225.46.35 = 97-1
priMR 130	TCCTTCTCTAACCTTCGAGCC	genotyping	SALK_038225.46.35 = 97-1
priMR 131	TGTATTTTCATGGTGTGTGGTTG	genotyping	GABI_243D08 = 97-2
priMR 132	TCCTTCTCTAACCTTCGAGCC	genotyping	GABI_243D08 = 97-2
priMR 133	TGAGCTAATTTGGTAGGTGGG	genotyping	SAIL_330_H06 = 97-3
priMR 134	TAATGGCTTTGGTTTATCCGG	genotyping	SAIL_330_H06 = 97-3
priMR 139	tctgcagattatccgtgagaaa	genotyping	SALK_112762.41.75 = 43-1 **(replacing priMR 119)**
priMR 140	ACCCTAACTTGCAGTGCTCA	genotyping	SALK_039929.55.25 = 79-1 **(replacing priMR 127)**
priMR 165	CAAAGGAACACTCTCATTCGC	genotyping	SALK_067511 & SALK_067639 = 73-1 & 73-2
priMR 166	TCCAACGTCTACGTCTACCTTTC	genotyping	SALK_067511 & SALK_067639 = 73-1 & 73-3

priMR 170	GAAACCAGACTCAAGCTGTGC	genotyping	SALK_010358.23.30 = 71-1
priMR 171	TTTGCGTAGAAAGCACTGTCC	genotyping	SALK_010358.23.30 = 71-1
priMR 172	GGCTGCAATAAAGAGGGAAAC	genotyping	SALK_152374.35.55 = 71-2
priMR 173	AGCAAAGTTGACAGCTTCAGG	genotyping	SALK_152374.35.55 = 71-2
priMR 174	TGGTTAGCCGTTAAAACGAAG	genotyping	GABI_054H02 = 79-2
priMR 175	TGTTGCTAAGTCGCATCTGTG	genotyping	GABI_054H02 = 79-2
priMR 180	TGGAAACTTTTTATTGCGGG	genotyping	SALK_034196.49.40 = 83-1
priMR 181	GCGCTACCATCTGCATCTTAC	genotyping	SALK_034196.49.40 = 83-1
priMR 182	GTAAGATGCAGATGGTAGCGC	genotyping	FLAG_148A09 = 83-2
priMR 183	TCTGCAGGTTTGACCATTACC	genotyping	FLAG_148A09 = 83-2
priMR 184	AACCGTAAACCCGACATCTTC	genotyping	SALK_039162.21.10 = 89-1
priMR 185	TACCAGAAACTCCATCAACGG	genotyping	SALK_039162.21.10 = 89-1
LBb1.3	ATTTTGCCGATTTCGGAAC	genotyping	genotyping SALK lines
GBF_AC161_LB1	ATATTGACCATCATACTCATTGC	genotyping	genotyping GABI-Kat lines
FL_LB4	CGTGTGCCAGGTGCCCACGGAATAGT	genotyping	genotyping FLAG lines
Syg_LB1	GCCTTTTCAGAATGGATAAATAGCCTTGCTTCC	genotyping	genotyping SAIL lines
priMR 141	aaaaagcaggctTCCAAGCAATGAGCGAAA	cloning	LP + attB1 for **AT1G29660** amplifying 2461bp promoter
priMR 142	agaaagctgggtCGATCTCACAAAACAAAAC	cloning	RP + attB2 for **AT1G29660** amplifying 2461bp promoter
priMR 143	aaaaagcaggctGGGCCCGTACTAAACATCC	cloning	LP + attB1 for **AT3G21500** amplifying 1441bp promoter
priMR 144	agaaagctgggtCCAACGTATATATAGTGG	cloning	RP + attB2 for **AT3G21500** amplifying 1441bp promoter
priMR 145	aaaaagcaggctCAAATGGAGATGATGATTGGA	cloning	LP + attB1 for **AT5G62210** amplifying 991bp promoter
priMR 146	agaaagctgggtTGTTGTCTTCTTGAAAAGAG	cloning	RP + attB2 for **AT5G62210** amplifying 991bp promoter
priMR 147	aaaaagcaggcttcgggactttttacattttgaa	cloning	LP + attB1 for **AT2G47115** amplifying 480bp promoter
priMR 167	agaaagctgggtCGATCAATGCC*G*Tgattt	cloning	RP + attB2 for **AT2G47115** amplifying 480bp promoter
priMR 151	aaaaagcaggctgttggcctacggcacttg	cloning	LP + attB1 for **AT1G72260** amplifying 357bp promoter
priMR 152	agaaagctgggtcttttgaatggtttacttg	cloning	RP + attB2 for **AT1G72260** amplifying 357bp promoter
priMR 153	aaaaagcaggcttccgttgttgaatttgtcg	cloning	LP + attB1 for **AT3G20520** amplifying 826bp promoter
priMR 154	agaaagctgggtggtagtggagagctttca	cloning	RP + attB2 for **AT3G20520** amplifying 826bp promoter
priMR 157	aaaaagcaggcttggtatgcaatccctcca	cloning	LP + attB1 for **AT3G26790** amplifying 2220bp promoter
priMR 158	agaaagctgggtctctctcaattggttaac	cloning	RP + attB2 for **AT3G26790** amplifying 2220bp promoter
priMR 159	aaaaagcaggctacttgccatcggtcttgg	cloning	LP + attB1 for **AT2G17710** amplifying 858bp promoter
priMR 160	agaaagctgggtaccacaacttcacctttc	cloning	RP + attB2 for **AT2G17710** amplifying 858bp promoter
ACT11_F	AACTTTCAACACTCCTGCCATG	RT-PCR control gene	amplification of *ACTIN 11* transcripts
ACT11_R	CTGCAAGGTCCAAACGCAGA	RT-PCR control gene	amplification of *ACTIN 11* transcripts
WOX9_F	ccatcaacttcggaccagctt	RT-PCR control gene	amplification of *WOX9* transcripts
WOX9_R	tccctcacattgaacggtcct	RT-PCR control gene	amplification of *WOX9* transcripts
TT10 FOR	ATACATCACACAATGCCCGATTC	RT-PCR control gene	LP amplifying seed-coat-specific gene *TT10*
TT10 REV	CCTCCTGTCCTTACGAACTCC	RT-PCR control gene	RP amplifying seed-coat-specific gene *TT10*
priMR 186	TCAATGACCAGGTTCGAGTG	RT-PCR control gene	LP amplifying seed-coat-specific gene *AT5G42530*
priMR 187	CAAGAGGAAGCCCAGAAACA	RT-PCR control gene	RP amplifying seed-coat-specific gene *AT5G42530*
FWA FOR	GTCACATCCACCAACTCTACCAG	RT-PCR control gene	LP amplifying endosperm-specific gene *FWA*
FWA REV	GCCACTTGTCCACCGAAGG	RT-PCR control gene	RP amplifying endosperm-specific gene *FWA*
AGL46 FOR	ATGGCAACAACTGTAGTAGATGAAG	RT-PCR control gene	LP amplifying endosperm-specific gene *AGL46*
AGL46 REV	TTGGTTTGATGGGATTATGGACTG	RT-PCR control gene	RP amplifying endosperm-specific gene *AGL46*
AGL62 FOR	CCTCCTCACCAACACAACAAC	RT-PCR control gene	LP amplifying endosperm-specific gene *AGL62*
AGL62 REV	AGATAACGCAAGTTCCTCAACG	RT-PCR control gene	RP amplifying endosperm-specific gene *AGL62*
